# Identification of a noncanonical function for ribose-5-phosphate isomerase A promotes colorectal cancer formation by stabilizing and activating β-catenin via a novel C-terminal domain

**DOI:** 10.1371/journal.pbio.2003714

**Published:** 2018-01-16

**Authors:** Yu-Ting Chou, Jeng-Kai Jiang, Muh-Hwa Yang, Jeng-Wei Lu, Hua-Kuo Lin, Horng-Dar Wang, Chiou-Hwa Yuh

**Affiliations:** 1 Institute of Molecular and Genomic Medicine, National Health Research Institutes, Zhunan, Miaoli, Taiwan; 2 Institute of Biotechnology, National Tsing-Hua University, Hsinchu, Taiwan; 3 Division of Colon and Rectal Surgery, Department of Surgery, Taipei Veterans General Hospital, Taiwan; 4 Institute of Clinical Medicine, National Yang-Ming University, Taipei, Taiwan; 5 Department of Life Sciences, National Central University, Jhongli City, Taoyuan, Taiwan; 6 Institute of Bioinformatics and Structural Biology, National Tsing-Hua University, Hsinchu, Taiwan; 7 Department of Biological Science and Technology, National Chiao Tung University, Hsinchu, Taiwan; University of California at Los Angeles, United States of America

## Abstract

Altered metabolism is one of the hallmarks of cancers. Deregulation of ribose-5-phosphate isomerase A (RPIA) in the pentose phosphate pathway (PPP) is known to promote tumorigenesis in liver, lung, and breast tissues. Yet, the molecular mechanism of RPIA-mediated colorectal cancer (CRC) is unknown. Our study demonstrates a noncanonical function of RPIA in CRC. Data from the mRNAs of 80 patients’ CRC tissues and paired nontumor tissues and protein levels, as well as a CRC tissue array, indicate RPIA is significantly elevated in CRC. RPIA modulates cell proliferation and oncogenicity via activation of β-catenin in colon cancer cell lines. Unlike its role in PPP in which RPIA functions within the cytosol, RPIA enters the nucleus to form a complex with the adenomatous polyposis coli (APC) and β-catenin. This association protects β-catenin by preventing its phosphorylation, ubiquitination, and subsequent degradation. The C-terminus of RPIA (amino acids 290 to 311), a region distinct from its enzymatic domain, is necessary for RPIA-mediated tumorigenesis. Consistent with results in vitro, RPIA increases the expression of β-catenin and its target genes, and induces tumorigenesis in gut-specific promotor-carrying RPIA transgenic zebrafish. Together, we demonstrate a novel function of RPIA in CRC formation in which RPIA enters the nucleus and stabilizes β-catenin activity and suggests that RPIA might be a biomarker for targeted therapy and prognosis.

## Author summary

The pentose phosphate pathway generates NADPH, pentose, and ribose-5-phosphate by RPIA for nucleotide synthesis. Deregulation of RPIA is known to promote tumorigenesis in liver, lung, and breast tissues; however, the molecular mechanism of RPIA-mediated CRC is unknown. Here, we demonstrate a role of RPIA in CRC formation distinct from its role in these other tissues. We showed that RPIA is significantly elevated in CRC. RPIA increased cell proliferation and oncogenicity via activation of β-catenin, with RPIA entering the nucleus to form a complex with APC and β-catenin. Further investigation suggested that RPIA protects β-catenin by preventing its phosphorylation, ubiquitination, and subsequent degradation. In addition, the C-terminus of RPIA (amino acids 290 to 311), a portion of the protein not previously characterized, is necessary for RPIA-mediated tumorigenesis. Finally, we observed that transgenic expression of RPIA increases the expression of β-catenin and its target genes and induces tumorigenesis. Our findings suggest that RPIA can enter the nucleus and associate with APC/β-catenin, and suggest precise treatment of human CRC by targeting its nonenzymatic domain.

## Introduction

Colorectal cancer (CRC) is one of the most common forms of cancers and results in more than 600,000 deaths annually [1–3]. Mutations in adenomatous polyposis coli (APC) and β-catenin, members of the Wnt signaling cascade, are among the major causes of colon tumorigenesis [4–6]. APC acts as a cytoplasmic scaffolding protein and induces the ubiquitin-mediated degradation of β-catenin [7]. In addition to its cytoplasmic activity, APC also modulates nuclear β-catenin levels as a result of its intrinsic nuclear-cytoplasmic shuttling capability [8–11]. Truncation of APC protein results in accumulation of nuclear β-catenin in CRC cells [12–14]. However, existing APC truncation mutants differentially affect the phosphorylation and ubiquitination of β-catenin, suggesting that these functions may be controlled by different APC domains [15,16]. In the nucleus, β-catenin acts as a coactivator with T-cell transcription factor 4 (Tcf-4)/lymphocyte enhancement factor (LEF) to activate the transcription of downstream targets such as *Cyclin D1* (*CCND1*) and *Cyclin E2* (*CCNE2*). Abnormal activation of the β-catenin signaling pathway can lead to increased cell proliferation and immortalization [17–19]. For example, a human CRC cell line expressing wild-type (WT) APC and a mutant version of β-catenin protein (with a single amino acid deletion at residue S45) is sufficient to induce a cancerous phenotype [20]. However, the precise activation process of β-catenin signaling is still largely unknown.

The pentose phosphate pathway (PPP) is critical for cancer cell survival and proliferation [21,22]. Ribose-5-phosphate isomerase A (RPIA) is an important integral member of the PPP and regulates cancer cell growth and tumorigenesis [2,23,24]. In pancreatic ductal adenocarcinoma (PDAC), RPIA expression is required for maintenance of tumor cells overexpressing KRas^G12D^, an activated form of Ras [23]. Our previous study showed that in hepatocellular carcinoma (HCC), RPIA regulates tumorigenesis via PP2A and extracellular signal-regulated kinase (ERK) signaling [24]. Studies performed in colon tumor tissues expressing microRNA-124 revealed that cells expressing low RPIA levels led to a reduced tumor size, while high RPIA expression was correlated with reduced survival and increased tumor growth [2].

Here, we report that in CRC tissue RPIA is significantly up-regulated, and it is expressed at multiple stages of tumorigenesis, including early stages. It directly interacts with β-catenin and APC to activate target genes downstream of β-catenin that are important for carcinogenesis. High levels of RPIA expression stabilize β-catenin levels by preventing phosphorylation and ubiquitination of β-catenin. Transgenic zebrafish overexpressing RPIA under the control of a gut-specific promoter exhibited enhanced β-catenin expression and elevated mRNA levels of the colon cancer marker gene *ccne1*. Our work uncovers a new role of RPIA and provides a molecular mechanism of RPIA-mediated β-catenin stabilization and activation necessary for colon cancer formation.

## Results

### RPIA is highly expressed in different stages of human CRC tissue

**Fig 1:**
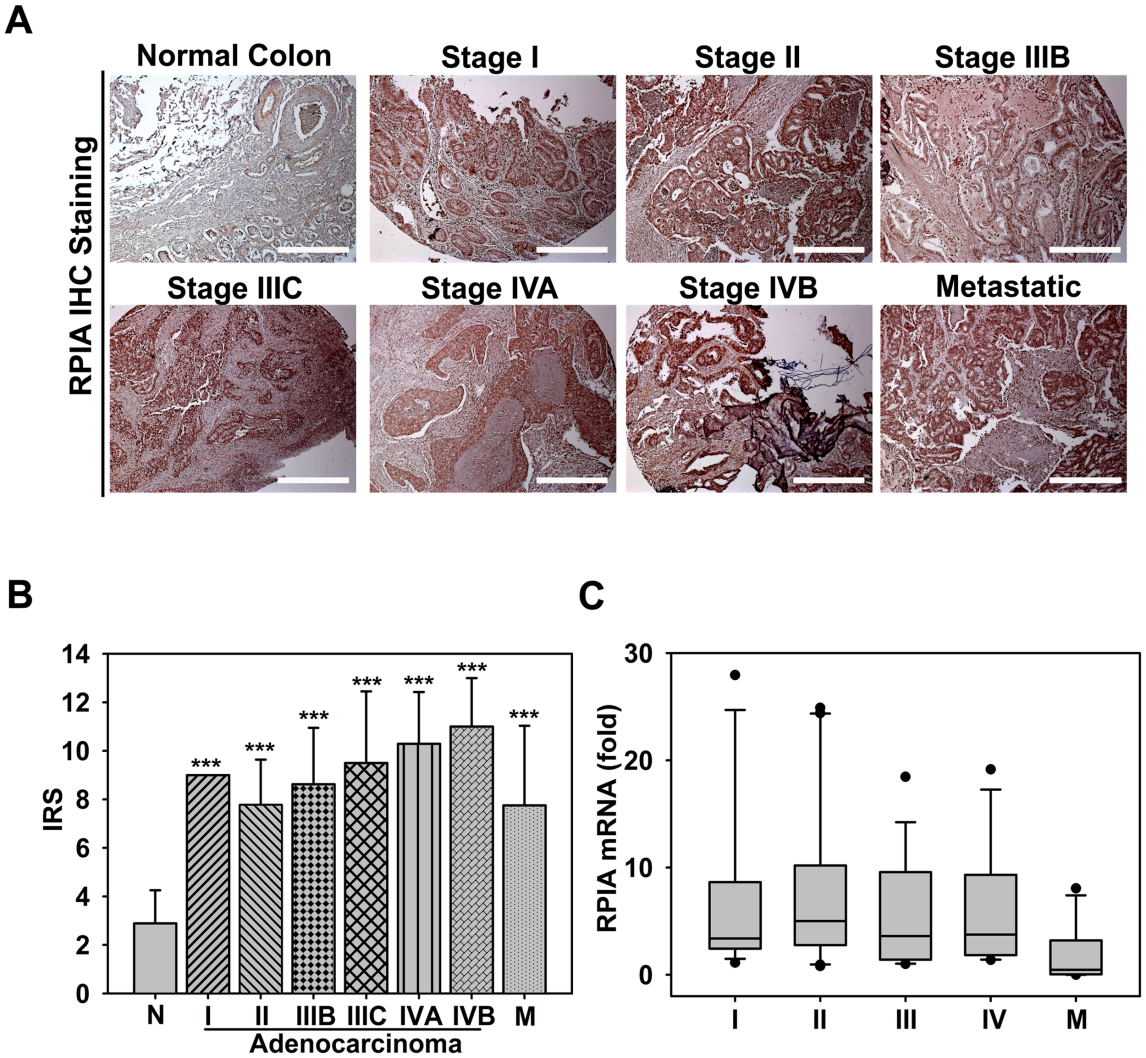
RPIA is highly expressed in different stages of CRC. (A) Representative RPIA IHC staining at different stages of colon cancer is shown. Scale bar: 500 μm. (B) The average IRS for RPIA staining showed significantly increased RPIA expression from stage I to IVB and the M. Stage III was divided into IIIB and IIIC, and stage IV was divided into IVA and IVB based on their subcategories. The statistical significance was calculated with Student *t* test (*** *P* < 0.001). (C) The average RPIA mRNA fold change in paired tissues (tumor tissue versus the surrounding normal tissue) from CRC patients at stages I to IV. The box plot indicates the median (central horizontal line), 75th percentile (the top of box), 25th percentile (the bottom of box), maximum value (the top end), minimum value (the bottom end), and the outlier (the point). Data can be found in S1 Data. CRC, colorectal cancer; IHC, immunohistochemistry; IRS, immunoreactive score; M, metastasis stage; N, normal colorectal tissue; RPIA, ribose-5-phosphate isomerase A.

To assess the role of RPIA in the progression of CRC, we measured RPIA protein levels using immunohistochemistry (IHC) with tissue arrays from stage I through IVB and metastatic colon cancer. The RPIA immunoreactive score (IRS) was calculated by multiplying the staining intensity by the proportion of positive cells [25] (S1A Fig). Highly elevated RPIA expression was found in all stages of colon cancer when compared to non-cancerous samples (Fig 1A). A control, non-immune antibody was employed to assess background staining and was stained negative. In Fig 1A, “normal colon” shows dark staining in an epithelial region to the lower left that is of much lower intensity than that of the tumor samples. IRS quantification revealed that RPIA expression is significantly up-regulated in all stages of colon adenocarcinoma and even in metastatic carcinoma (Fig 1B). To examine whether the RPIA mRNA level is also up-regulated in CRC patients, 80 paired tissues, including tumors and the adjacent normal tissues, were analyzed using real-time quantitative PCR (qPCR). In 78% (62 of 80) of the CRC specimens, RPIA mRNA was more than 2-fold overexpressed from stage I to IV and in metastatic carcinoma (Fig 1C). Taken together, we found that RPIA is overexpressed at both the mRNA and protein levels in all stages of colon cancer formation.

### RPIA levels are positively correlated with β-catenin protein levels, cellular proliferation, and oncogenicity

**Fig 2:**
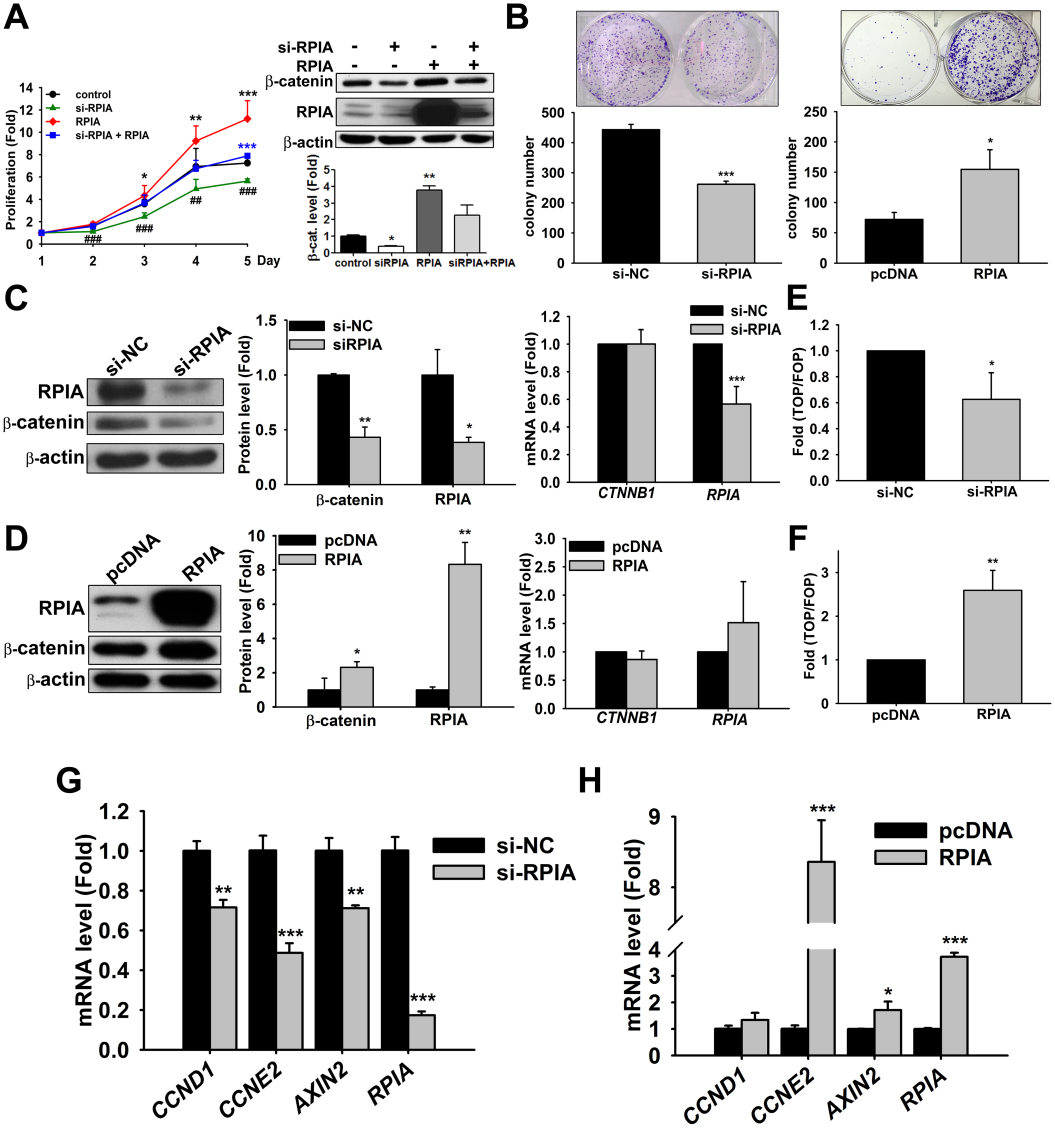
RPIA regulates colon cell proliferation through β-catenin expression in HCT116 cells. (A) Knockdown of RPIA significantly reduced cell proliferation and RPIA overexpression enhanced cell proliferation in HCT116 cells. Co-treatment of si-RPIA and pcDNA-RPIA rescued the reduction of cellular proliferation upon knockdown of RPIA in HCT116. Cell viability assays were performed by measuring the cells at the second, third, fourth, and fifth days and the proliferation fold is compared to control cell at the first day. Control: Co-transfect with scramble RNA and pcDNA empty vector as negative control. (B) RPIA knockdown significantly reduced colony formation ability, and RPIA overexpression enhanced colony formation ability in HCT116 cells. si-NC: Transfect with scramble siRNA as negative control. Representative images of colonies were shown on top of the quantification result. (C) Knockdown of RPIA reduced β-catenin protein levels as measured by western blotting (left panel) and quantified using Image J (middle panel) but did not significantly alter mRNA levels of β-catenin as measured by qPCR (right panel) in HCT116 cells. (D) RPIA overexpression increased β-catenin protein levels (left and middle panels) but did not affect β-catenin mRNA levels (right panel) in HCT116 cells. (E) Knock down of RPIA reduced the β-catenin/TCF luciferase reporter activity in HCT116 cells. (F) Overexpression of RPIA induced the β-catenin/TCF luciferase reporter activity in HCT116 cells. (G) Knockdown of RPIA decreased the mRNA levels of the β-catenin target genes *CCND1*, *CCNE2*, and *AXIN2* in HCT116 cells. (H) Overexpression of RPIA increased the mRNA levels of the β-catenin target genes *CCND1*, *CCNE2*, and *AXIN2* in HCT116 cells. The statistical significance was calculated with Student *t* test (* 0.01 < *P* < 0.05, ** 0.001 < *P* < 0.01, and *** *P* < 0.001). Data can be found in S2 Data. *AXIN2*, *Axis inhibition protein 2*; *CCND1*, *Cyclin D1*; *CCNE2*, *Cyclin E2*; *CTNNB1*, *CATENIN BETA 1*; pcDNA, pcDNA3 vector control; qPCR, quantitative PCR; RPIA, ribose-5-phosphate isomerase A; si-NC, negative control small interfering RNA; si-RPIA, RPIA small interfering RNA; TCF, T-cell transcription factor.

To examine the effects of RPIA overexpression on cellular proliferation, two colon cancer cell lines, HCT116 and SW480, were used. SW480 is a human colon cancer cell line with APC C-terminal truncation at 1338, but the β-catenin binding region is retained; HCT116 is a highly metastatic cell line with WT APC and both an S45 mutation and the WT allele for β-catenin, but most of the β-catenin protein comes from the mutant allele. WST-1 assays examine metabolic activity that represent the viability of the cell. We tested three small interfering RNAs (siRNAs) (S1B Fig), which were pooled or treated separately, and found all three siRNAs had similar effects. Therefore, we selected number 3 siRNA for the rest of the experiments. Knock down of RPIA significantly decreased cell proliferation in both cell lines (Fig 2A and S2A Fig). Conversely, overexpression of RPIA increased cell proliferation in both HCT116 and SW480 cells (Fig 2A and S2A Fig). In the knockdown and followed by overexpressing the RPIA for rescue, the results clearly showed that RPIA small interfering RNA (si-RPIA) decreased proliferation and β-catenin protein level can be rescued by overexpression RPIA in both HCT116 and SW480 cell lines (Fig 2A and S2A Fig). Knockdown of RPIA also dramatically decreased the colony formation ability in both cell lines (Fig 2B and S2B Fig), and overexpression of RPIA increased the colony formation ability in both HCT116 and SW480 cells (Fig 2B and S2B Fig). These data suggest that the RPIA expression level is positively correlated with cellular proliferation and colony formation ability in colon cancer cells.

Aberrant β-catenin accumulation is a major cause of uncontrollable proliferation in colon cancer cells [26,27]. β-catenin exerts its proliferation-promoting effects via translocation to the nucleus where it binds to T-cell transcription factor (TCF)/LEF to activate the transcription of downstream β-catenin target genes [28]. Therefore, we were interested in determining whether RPIA affects the β-catenin level in colon cancer cells using HCT116 and SW480 cells.

Our results indicate that knockdown of RPIA decreased the β-catenin protein level in both HCT116 (Fig 2C) and SW480 cells (S2C Fig) without affecting β-catenin (encoded by *CTNNB1* gene) mRNA levels. Conversely, RPIA overexpression increased β-catenin protein expression levels in both HCT116 (Fig 2D) and SW480 cell lines (S2D Fig), while β-catenin mRNA levels were unchanged. Using TOPflash/FOPflash luciferase reporter assay, we found that β-catenin activity was significantly attenuated upon RPIA knockdown (Fig 2E and S2E Fig) and dramatically increased when RPIA was overexpressed (Fig 2F and S2F Fig). Using qPCR to detect the expression levels of known downstream targets of β-catenin, including *CCND1*, *CCNE2*, and *AXIN2*, we found that knockdown and overexpression of RPIA reduced (Fig 2G and S2G Fig) and increased (Fig 2H and S2H Fig), respectively, the expression of these target genes. However, the effect of overexpression of RPIA was not as dramatic as that of knock down of RPIA, likely because CRC cell lines already overexpress RPIA. These results suggest that the RPIA expression level is positively correlated with β-catenin protein levels and its transcriptional activity.

### RPIA-mediated stabilization of β-catenin in colon cancer cells does not involve ERK signaling

**Fig 3:**
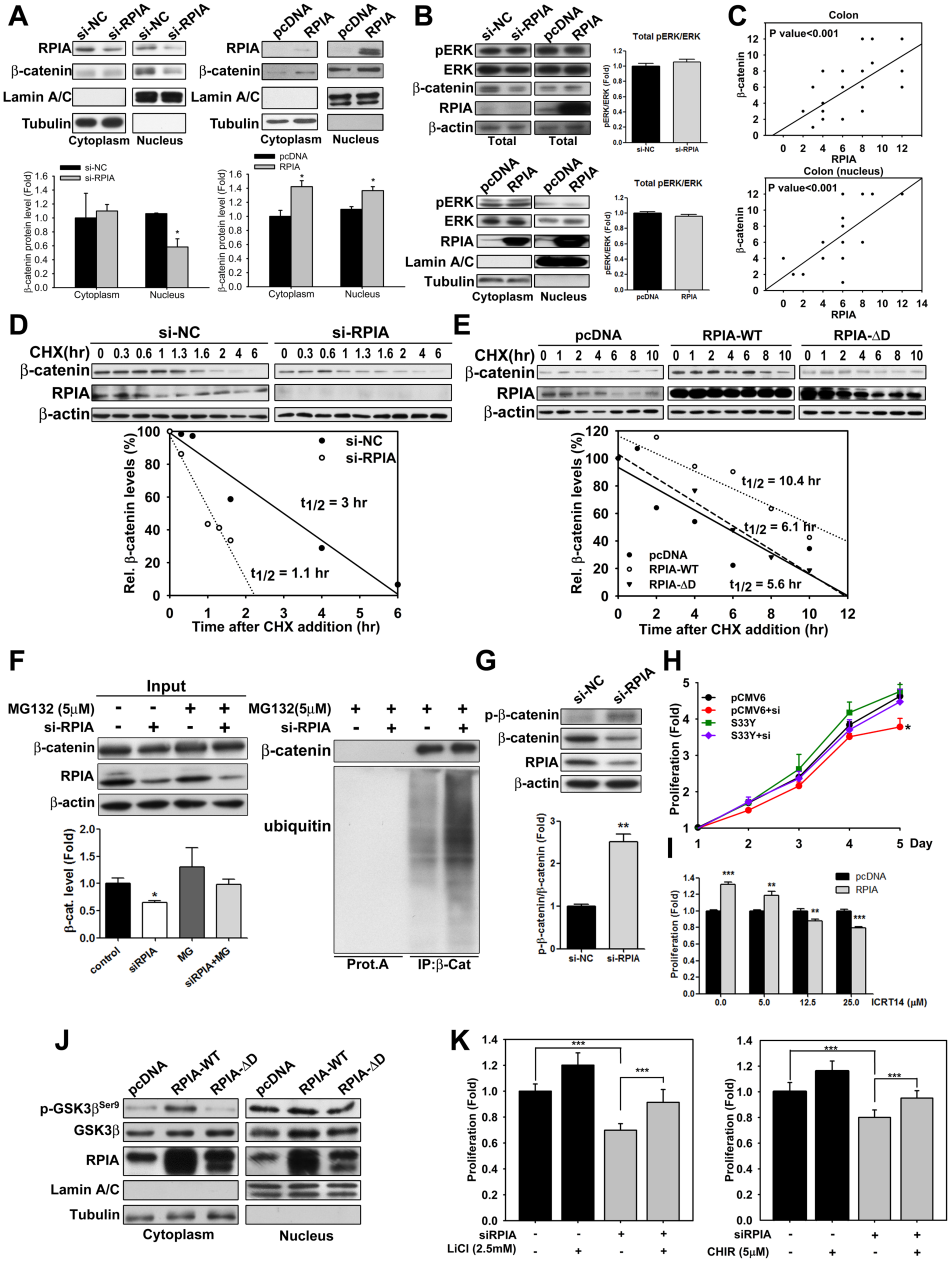
RPIA expression is positively correlated with β-catenin protein levels and stability in HCT116 cells. (A) Knockdown of RPIA reduced β-catenin protein levels and overexpression of RPIA increased β-catenin protein levels in both the cytoplasmic and nuclear fractions of HCT116 cells. (B) Knockdown of RPIA did not decrease ERK and pERK protein levels, which were measured by western blotting in total protein analysis (up panel) in HCT116. Conversely, overexpression of RPIA did not increase ERK and pERK protein levels (up panel). In the lower panel, both cytoplasmic and nuclear fraction showed that ERK and pERK protein levels did not up-regulate in HCT116. (C) Scatter plots show a positive correlation between RPIA and β-catenin expression in the colon tissue or nucleus. (D) To determine the half-life of β-catenin protein, western blots were used to measure the abundance of β-catenin at different time points following the addition of 10 μg/ml of the protein synthesis inhibitor CHX to HCT116 cells transfected with either control siRNA or RPIA-siRNA. The lower panels show plots of the relative β-catenin protein level, expressed as a percentage as a function of time after CHX treatment. (E) RPIA-ΔD lost the ability to stabilize β-catenin. Relative β-catenin protein levels as measured by quantification of western blot are shown in HCT116 cells. (F) The reduced β-catenin levels by RPIA knockdown were rescued by 5 μM of MG132 treatment (left panel). Inhibition of RPIA stimulated ubiquitination of β-catenin (right panel). β-Catenin was precipitated by specific antibody. Coprecipitated ubiquitin levels were examined via western blot with antiubiquitin antibody. (G) Phosphorylated β-catenin (at Ser33/Ser37) versus total β-catenin was elevated upon RPIA knockdown. Gel images are shown in the up panel. (H) Overexpression of nondegradable β-catenin can overcome the growth inhibition induced by RPIA knockdown in HCT116 cells. The proliferation fold is compared to pMCV6 transfected control cell at first day. (I) The elevated viability induced by expression of RPIA was decreased upon ICRT14 (β-catenin inhibitor) treatment. Dose-dependent effects were revealed in HCT116 cells. (J) pGSK3β^Ser9^ protein expression levels were up-regulated in the cytoplasmic extract upon overexpression of RPIA-WT but not upon RPIA-ΔD in HCT116 cells. (K) Cell proliferation was measured in RPIA knockdown HCT116 cells combined with 2.5 mM LiCl or 5 μM CHIR99021, respectively. The statistical significance was calculated with the Student *t* test (*** *P* < 0.001). Data can be found in S3 Data. CHX, cycloheximide; ERK, extracellular signal-regulated kinase; LiCl, lithium chloride; MG132, proteasome inhibitor; pcDNA, pcDNA3 vector control; pERK, phosphorylated-ERK; Rel, relative; RPIA-ΔD, RPIA deletion domain D mutant; RPIA, ribose-5-phosphate isomerase A; RPIA-WT, RPIA wild type; si-NC; negative control siRNA; siRNA, small interfering RNA; si-RPIA, RPIA small interfering RNA.

Our previous study indicates that ERK signaling participates in RPIA-mediated hepatocarcinogenesis [24]. These observations, in combination with other studies demonstrating crosstalk between β-catenin and ERK in other types of tumors [29,30], led us to investigate whether ERK signaling might also play a role in RPIA-mediated tumorigenesis in colon cancer. Therefore, the effects of both RPIA overexpression and reduction on both β-catenin and ERK protein levels were examined in HCT116 and SW480 cells. Reduction of RPIA by knockdown significantly decreased nuclear β-catenin protein levels in HCT116 (Fig 3A) and both cytoplasmic and nuclear β-catenin protein were decreased in SW480 (S3A Fig), but did not affect the levels of activated, phosphorylated ERK (pERK) (Fig 3B and S3B Fig). Conversely, overexpression of RPIA increased β-catenin protein levels in HCT116 (Fig 3A) and SW480 (S3A Fig) cells without altering both cytoplasmic and nuclear level of pERK and ERK levels in these cell lines (Fig 3B and S3B Fig). Other mechanisms important for intestinal cell proliferation such as epidermal growth factor receptor (EGFR) signaling were also examined. Neither EGFR protein level nor the phosphorylated EGFR (pEGFR) were altered upon overexpression or knockdown RPIA in both cell lines (S3C Fig). Furthermore, IHC staining analyses revealed that both RPIA and β-catenin protein levels were significantly higher in the nuclei of colon cancer tissues than in the nuclei of normal tissues. In addition, a positive correlation was presented between RPIA and β-catenin protein levels in the nuclei of colon cancer tissue (Fig 3C). These data suggest that promotion of β-catenin signaling, but not ERK or EGFR signaling, is involved in transducing the effects of RPIA-mediated colon cancer tumorigenesis.

As only β-catenin protein levels were affected by the levels of RPIA expression, we proposed that RPIA increased β-catenin protein stability in colon cancer cells. To test this hypothesis, the protein synthesis inhibitor cycloheximide (CHX) was used to determine the half-life of β-catenin in cell lines with either reduced or elevated levels of RPIA. Reduction of RPIA by knockdown decreased the β-catenin protein half-life from 3 to 1.1 h in HCT116 cells (Fig 3D) and from 2.7 to 0.9 h in SW480 cells (S3D Fig). Conversely, overexpression of RPIA strongly increased the half-life of β-catenin from 5.6 to 10.4 h in HCT116 cells (Fig 3E) and from 4.9 to 9.9 h in SW480 cells (S3E Fig). Therefore, we conclude that RPIA increases β-catenin protein stability in colon cancer cells.

Because it has been shown that β-catenin protein levels can be controlled by ubiquitination and subsequent proteasome degradation [31], we tested whether RPIA could modulate β-catenin protein levels in colon cancer cell lines by changing the ubiquitination-mediated degradation process. As shown previously, RPIA knockdown resulted in a reduction in β-catenin protein levels (Fig 3F and S3F Fig, left panel). Treatment with 5 μM MG132, a proteasome inhibitor, rescued the reduction in β-catenin protein levels observed in cells expressing RPIA siRNA (Fig 3F and S3F Fig, left panel). Immunoprecipitation (IP) revealed that more ubiquitin was coprecipitated with β-catenin in RPIA-siRNA-treated cells than in negative control siRNA (si-NC)-treated cells (Fig 3F and S3F Fig, right panel). In addition, the phosphorylated, targeted for degradation form of β-catenin (with phosphorylation at residues Ser33/Ser37) was elevated upon RPIA knockdown relative to total β-catenin (Fig 3G and S3G Fig). Moreover, expression of a non-degradable β-catenin mutant (S33Y) rescued the reduction of proliferation upon RPIA knockdown in HCT116 and SW480 cell lines (Fig 3H and S3H Fig). To demonstrate that β-catenin is indeed required downstream of RPIA, the β-catenin inhibitor ICRT14 was applied to the RPIA overexpression cells. The results showed that RPIA-promoted cellular proliferation was attenuated in a dose-dependent manner by ICRT14 (Fig 3I and S3I Fig). This confirms that β-catenin is required for RPIA overexpression-mediated cell proliferation. The levels of an inactive form of GSK3β (with phosphorylation at residue ser9; pGSK3β^ser9^), which does not have the ability to phosphorylate β-catenin, were further examined. Because phosphorylated GSK3β^ser9^ was elevated upon overexpression of RPIA in both HCT116 and SW480 cell lines and there is no difference in the nucleus GSK3β, we suggest the RPIA modulate GSK3β only in the cytoplasm (Fig 3J and S3J Fig). Moreover, treatment with GSK3β inhibitors (lithium chloride [LiCl] or CHIR99021) rescued the reduction of proliferation upon RPIA knockdown, indicating the involvement of GSK3β in this process (Fig 3K and S3K Fig). These data show that RPIA retains a novel function to protect β-catenin from phosphorylation-mediated ubiquitination and degradation via proteasomes.

### RPIA forms a complex with β-catenin and APC in both the nucleus and cytoplasm

**Fig 4:**
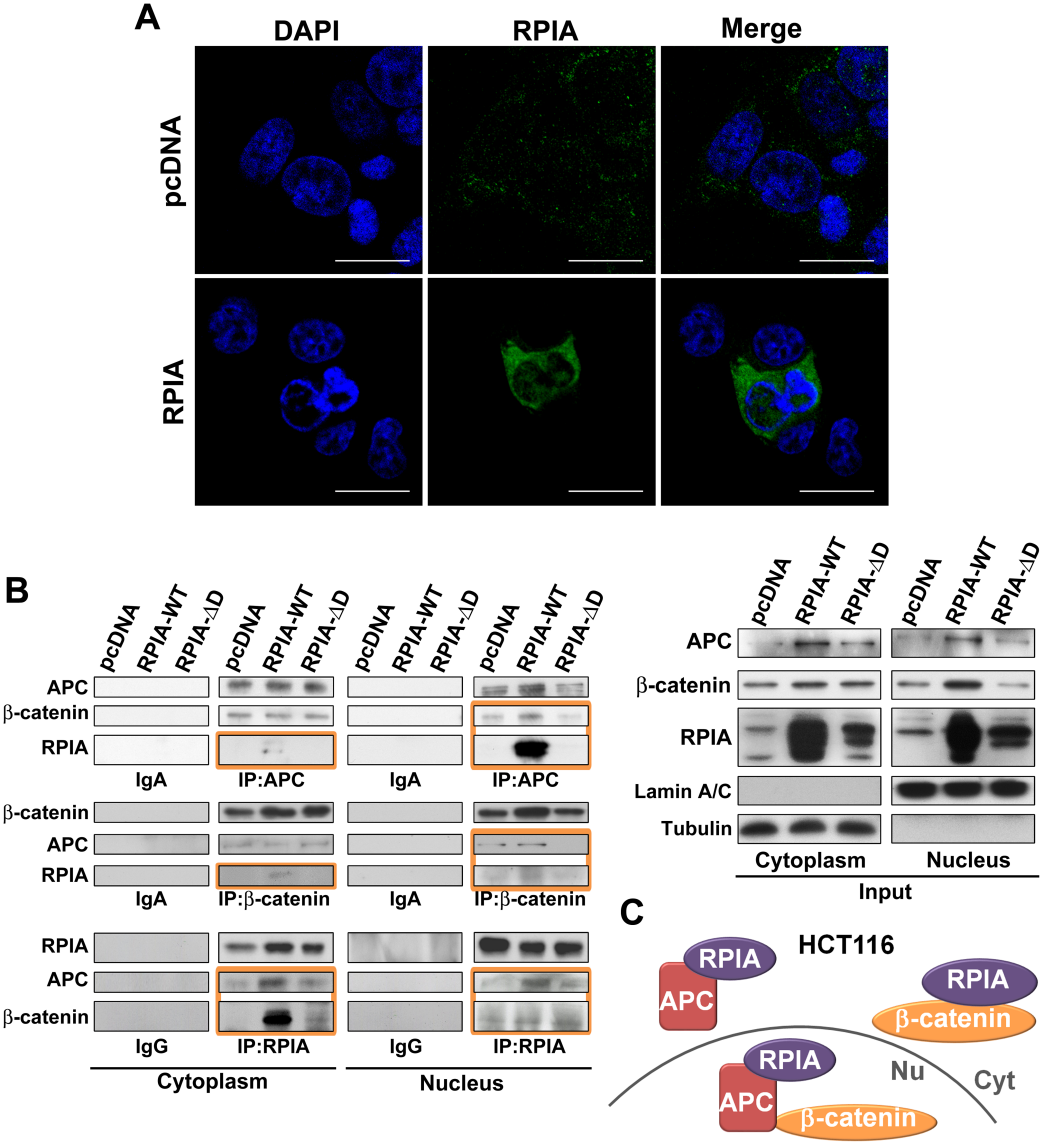
RPIA localizes in the nucleus and interacts with APC and β-catenin in HCT116 cells. (A) Nuclear localization of RPIA was detected by immunostaining with an anti-RPIA antibody (green) in HCT116 cells with and without overexpression of RPIA. DAPI was used to counterstain nuclei (blue). Scale bar: 50 μm. (B) Left panels: The cell lysates were precipitated by anti-APC, anti-β-catenin, and anti-RPIA antibodies in HCT116 cells. The APC, β-catenin, and RPIA interaction can be increased by RPIA-WT but not by RPIA-ΔD. Right panels: Protein loading input for IP assay of HCT116 cells. The orange boxes indicate the signals were enhanced by RPIA-WT but not in RPIA-ΔD. (C) Model of RPIA-β-catenin-APC interaction in HCT116 cell line. APC, adenomatous polyposis coli; Cyt, cytoplasm; IgA, immunoglobulin A; IP, immunoprecipitation; pcDNA, pcDNA3 vector control; Nu, nucleus; RPIA-ΔD, RPIA deletion domain D mutant; RPIA, ribose-5-phosphate isomerase A; RPIA-WT, RPIA wild type.

The cytoplasmic complex that targets β-catenin for degradation includes the scaffolding protein APC [32]. In addition, APC associates with β-catenin in the nucleus and directs the nucleocytoplasmic export of β-catenin [10,11]. We used immunostaining to detect RPIA localization. In the pcDNA3 vector only control (pcDNA), RPIA was expressed in the cytoplasm exclusively. Overexpression of RPIA in both HCT116 and SW480 cells resulted in an increase in nuclear and cytoplasmic RPIA expression (Fig 4A and S4A Fig) with a punctate pattern of RPIA in the nucleus of the DAPI-negative nucleoplasm [33]. IP of different proteins followed by western blotting indicated that RPIA can form a complex with APC and β-catenin in the nucleus in both HCT116 and SW480 cell lines (Fig 4B and 4C and S4B and S4C Fig). Interestingly, we noticed the minor difference between these cells. In HCT116, RPIA interacted strongly with APC and β-catenin, respectively, in the cytoplasm (Fig 4C). In addition, RPIA/APC and RPIA/β-catenin complex levels are induced by RPIA WT (RPIA-WT) (Fig 4B and 4C). In HCT116, the nuclear interaction of RPIA-β-catenin is much weaker than in cytoplasm. We suspect the interaction between RPIA and β-catenin in the nucleus might be indirectly through APC. In SW480, the nuclear RPIA-β-catenin interaction is much stronger than in cytoplasm. However, only the β-catenin bond to RPIA and promoted from RPIA-WT in cytoplasm in SW480 (S4B and S4C Fig) and the nuclear β-catenin-RPIA interaction can be regulated by the RPIA amount. The differences in HCT116 and SW480 might be caused by the truncated APC in SW480, and HCT116 has an S45 mutation from β-catenin.

### The C-terminal domain of RPIA is necessary for enhanced cell proliferation in colon cancer cells

**Fig 5:**
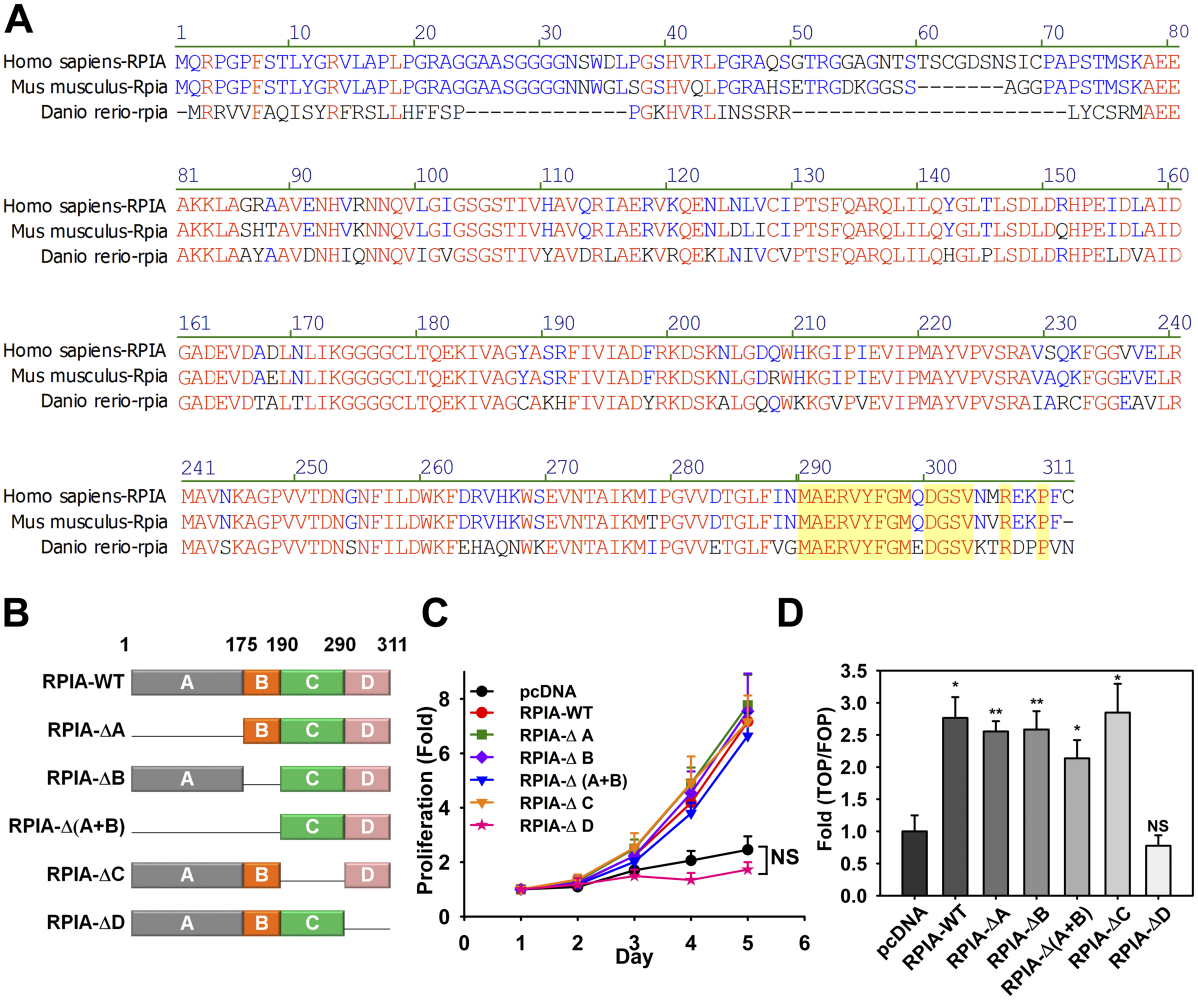
The C-terminal domain of RPIA containing amino acids 290 to 311 is required for cell proliferation and β-catenin stabilization in HCT116 cells. (A) The RPIA amino acid sequence in several species. Identical sequences are represented by red text. Blue text indicates >50% but <90% consensus. Yellow background indicates RPIA domain D. The analysis was conducted with Vector NTI-AlignX. (B) Schematic representation of RPIA and its deletion mutants. RPIA-WT; RPIA-ΔA, deletion of the A domain from amino acid 1 to 174 containing the isomerase active site in the N-terminus; RPIA-ΔB, deletion of the B domain from amino acid 175 to 189 containing the catalytic residue; RPIA-Δ (A+B), deletion of the A and B domains from amino acid 1 to 189; RPIA-ΔC, deletion of the C domain from amino acid 190 to 289; RPIA-ΔD, deletion of the D domain from amino acid 290 to 311 in the C-terminus of RPIA. (C) The effect on cell proliferation after the expression of RPIA-WT and the different RPIA deleted constructs in HCT116 cells. (D) RPIA-ΔD lost the ability to stimulate the TOPflash luciferase construct in HCT116 cells. The statistical significance was calculated with Student *t* test (* 0.01 < *P* < 0.05 and ** 0.001 < *P* < 0.01). Data can be found in S4 Data. NS, not significant; pcDNA, pcDNA3 vector control; RPIA, ribose-5-phosphate isomerase A; RPIA-WT, RPIA wild type.

The RPIA protein sequence is conserved among humans (*Homo sapiens*), mice (*Mus musculus*), and zebrafish (*Danio rerio*) (Fig 5A). To determine which protein domain(s) are important for RPIA-mediated tumor cell proliferation, five RPIA deletion mutants were generated. These include RPIA-ΔA (deletion of the active domain of RPIA), RPIA-ΔB (deletion of the catalytic domain of RPIA), RPIA-Δ(A+B), RPIA-ΔC, and RPIA deletion domain D mutant (RPIA-ΔD) (Fig 5B). The WT and five deletion mutants were transfected into HCT116 and SW480 cells. WST-1 assays were performed to examine metabolic activity, and the RNA and protein levels from the WT and five deletion mutants were verified (S5A and S5B Fig). We noticed that different RNA constructs might have different regulation of RPIA stability. Interestingly, only the expression of RPIA-ΔD failed to enhance cell proliferation, while the other mutants produced no significant changes in proliferation (Fig 4C and S4C Fig). These data suggest that the C-terminal domain D of RPIA (AAs 290 to 311) is essential for RPIA-mediated tumor cell proliferation. Domain D also seems to be necessary for the RPIA-mediated increase in β-catenin protein stability in colon cancer cells because overexpression of RPIA-ΔD did not stabilize β-catenin protein levels like the WT RPIA. Following overexpression of RPIA-ΔD, the half-life of β-catenin was approximately 6.1 and 3.3 h in HCT116 and SW480 cells, respectively, which was similar to that of the pcDNA vector alone (5.6 and 4.9 h in HCT116 and SW480 cells, respectively; Fig 3E and S3E Fig). Moreover, RPIA-ΔD did not interact with APC and β-catenin in either the cytoplasm or nucleus (Fig 4B and S4B Fig). Furthermore, RPIA-ΔD was unable to elevate TCF reporter activity in the colon cancer cells (Fig 5D and S5D Fig). These results demonstrate that domain D of RPIA is essential for the RPIA-mediated increase in β-catenin protein stability, activation of β-catenin target genes, and cell proliferation advantages seen in colon cancer cells. Together, our data also indicated that the RPIA D domain exhibits a novel function in addition to the enzymatic region.

### RPIA promotes intestinal tumorigenesis in *RPIA* transgenic zebrafish in vivo

**Fig 6:**
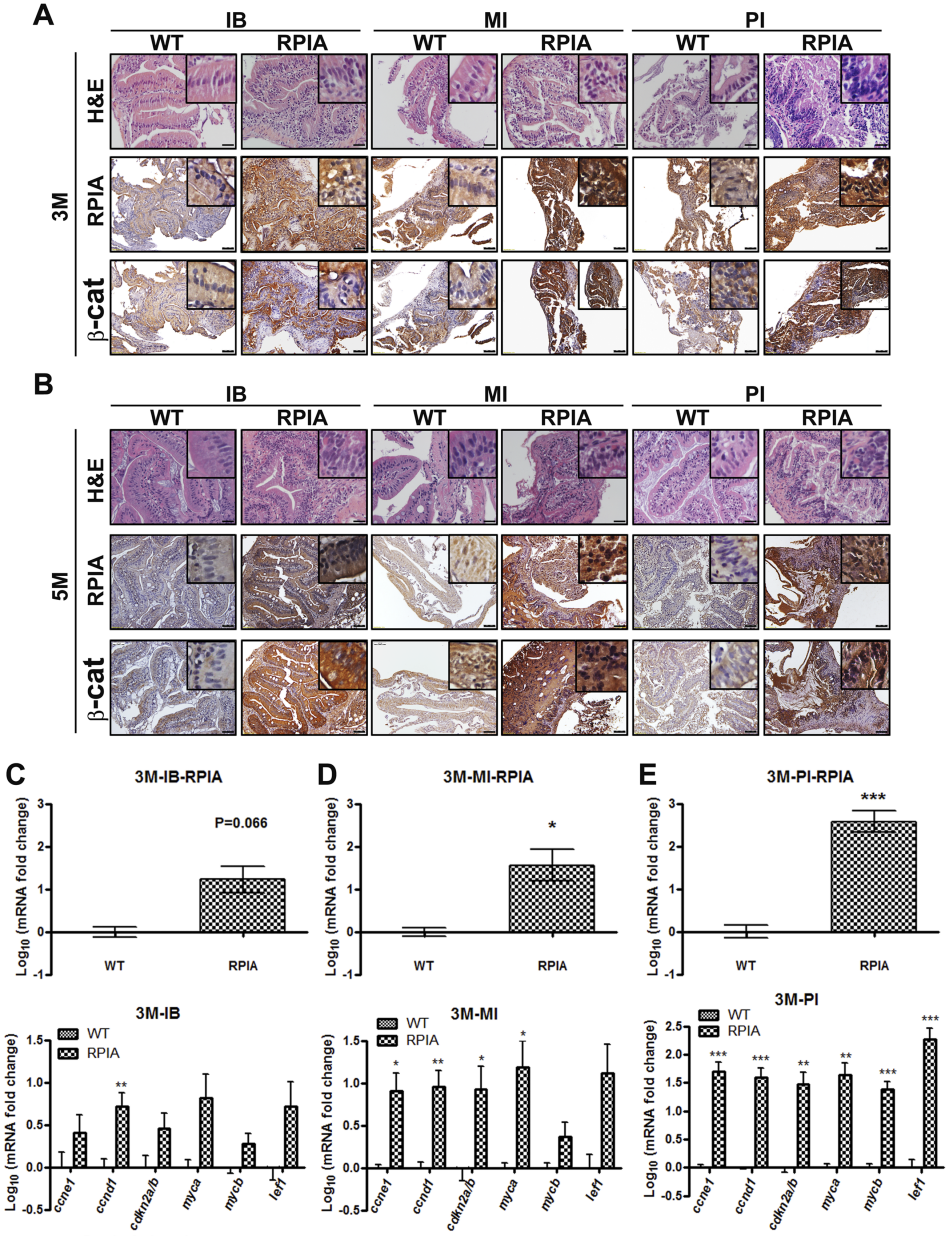
RPIA promotes β-catenin expression and tumorigenesis in vivo. (A and B) In the upper panels, the HE stain was examined in the AB line (WT) and in (A) 3M and (B) 5M Tg (*ifabp*:*RPIA*) zebrafish. As shown in the bottom panels, via IHC, β-catenin expression levels were increased in (A) 3M and (B) 5M Tg (*ifabp*:*RPIA*) fish as well as in different regions of the intestine: IB, MI, and PI. Magnification: 400X for HE and 200X for IHC. Scale bar: 20 μm for HE and 50 μm for IHC. (C-E) β-catenin target genes were elevated in 3M Tg (*ifabp*:*RPIA*) fish, especially in (E) the PI. The expression level of β-catenin target genes was analyzed in 3M control fish (*n* = 6) and RPIA Tg fish (*n* = 18) from three portions of guts. The gene expression levels were analyzed with qPCR. There are extreme data in each group, so they are removed for the statistical analysis. (C) For IB, the number of WT is 6, and the number for RPIA is 7. (D) For the MI, the number of WT is 6, and the number for RPIA is 13. (E) For PI, the number of WT is 3, and the number for RPIA is 9. The statistical significance was calculated with Student *t* test (* 0.01 < *P* < 0.05). Data can be found in S5 Data. 3M, 3-month-old; 5M, 5-month-old; *ccne1*, cyclin E1*; ccnd1*, cyclin D1; *cdkn2a/b*, cyclin-dependent kinase inhibitor 2a/b; HE, hematoxylin and eosin; IB, intestinal bulb; IHC, immunohistochemistry; *lef1*, lymphoid enhancer factor 1; MI, middle intestine; *myca*, oncogenes c-myca; *mycb*, oncogenes c-mycb; PI, posterior intestine; qPCR; quantitative PCR; RPIA, ribose-5-phosphate isomerase A; Tg, transgenic; WT, wild-type.

Histopathologically, many of the features found in human colon adenocarcinoma are similar to those in zebrafish, an important vertebrate cancer model system [34,35]. To test the effects of RPIA overexpression on colon cancer formation, we generated transgenic zebrafish that overexpressed RPIA under the control of a gut-specific promoter (*ifabp*). In particular, we analyzed the histopathology of the intestinal bulb (IB), middle intestine (MI), and posterior intestine (PI) collected from non-transgenic and transgenic (*ifabp*:*RPIA*) fish of different ages. Increased nuclear-to-cytoplasmic ratio, nuclear atypia, and moderately differentiated adenocarcinoma were observed in 3- and 5-month-old Tg (*ifabp*:*RPIA*) fish relative to the WT controls (Fig 6A and 6B, upper panel). In WT zebrafish, the β-catenin protein is detected at low levels in the intestinal villi at intracellular junctions [36]. Using IHC, we also observed low-level and intracellularly located β-catenin expression in WT fish, while overexpression of RPIA in transgenic fish resulted in increased β-catenin expression and nuclear localization (Fig 6A and 6B, lower panel).

We next explored the β-catenin target genes, including *ccne1*, *ccnd1*, *cdkn2a/b*, *myca*, *mycb*, and *lef1*. A log 10-fold change value was used to show the level of genes [37] in 3-month-old Tg (*ifabp*:*RPIA*) fish, and the expression levels of β-catenin target genes were found to be positively correlated with RPIA expression levels (Fig 6C and 6E) and more highly expressed in the PI (Fig 6E) than in the IB (Fig 6C). In addition, as *ccne1* is required for cell cycle/proliferation and tumor growth in CRC [29,38], it was used as colon tumorigenesis marker. The mRNA levels of *ccne1* were elevated in 3-month-old Tg (*ifabp*:*RPIA*) fish, especially in the PI, which had dramatically increased RPIA expression. In addition to 3-month-old fish, we also analyzed β-catenin target genes in 5-month-old fish (S6A–S6C Fig). In accordance with the 3-month-old Tg (*ifabp*:*RPIA*) fish, the β-catenin target genes were up-regulated more significantly in the PI than in the IB (S6C Fig). Then, we noticed that RPIA and the expression of most of the β-catenin target genes were slightly decreased in 5-month-old fish compared with 3-month-old fish. Whether this phenomenon was the result of mature recovery in zebrafish remains to be determined [37,39]. A number of physiological changes are associated with cancer in human patients [40,41] including decreased body weight, decreased body width, decreased body length, and reduced intestinal length. With the exception of reduced body length, all these changes were observed in transgenic 1-year-old fish (S6D–S6G Fig). These results demonstrate that in vivo, in an important model system, RPIA increases β-catenin protein levels and induces colon tumorigenesis, resulting in overall weaker and smaller fish.

## Discussion

Recent findings have revealed that the non-oxidative PPP is a critical pathway for tumor formation [21]. Aberrant activation of the canonical Wnt/β-catenin pathway has also been shown to be involved in gastrointestinal cancers [36]. In cancer cells, β-catenin protein has a dual function: at the membrane, β-catenin coordinates adherent junctions for maintenance of epithelial cell barriers, while in the nucleus, β-catenin acts as a transcriptional activator to regulate proliferation genes [42–44]. In this study, we demonstrate that RPIA exhibits a novel role in CRC through association with and activation of β-catenin. High levels of RPIA expression were detected early and throughout multiple stages of 80 paired samples in CRC human patients. These results are consistent with the Human Gene Database and the Human Protein Atlas, which indicates about the 10-fold higher expression of RPIA in CRC patients than in normal tissues. Furthermore, we found RPIA stabilizes and subsequently promotes activation of β-catenin downstream target genes. We suggest that the increased cellular proliferation and oncogenicity are induced by RPIA through β-catenin pathway.

In other cancer types, RPIA promotes tumorigenesis via different mechanisms [2, 23,24]. In pancreatic and hepatic cancers, RPIA expression is required for maintenance of tumor cells overexpressing KRas^G12D^, while in HCC, RPIA regulates tumorigenesis via PP2A and ERK signaling. Interestingly, the RPIA-mediated CRC tumorigenesis does not involve the activation of ERK and presumably Ras signaling. This observation raises an interesting question: "Is RPIA-mediated stabilization and activation of β-catenin merely in CRC?" If valid, it may influence the decision of choosing different therapeutic targets of molecules and/or signaling pathways for treating different cancer types.

**Fig 7:**
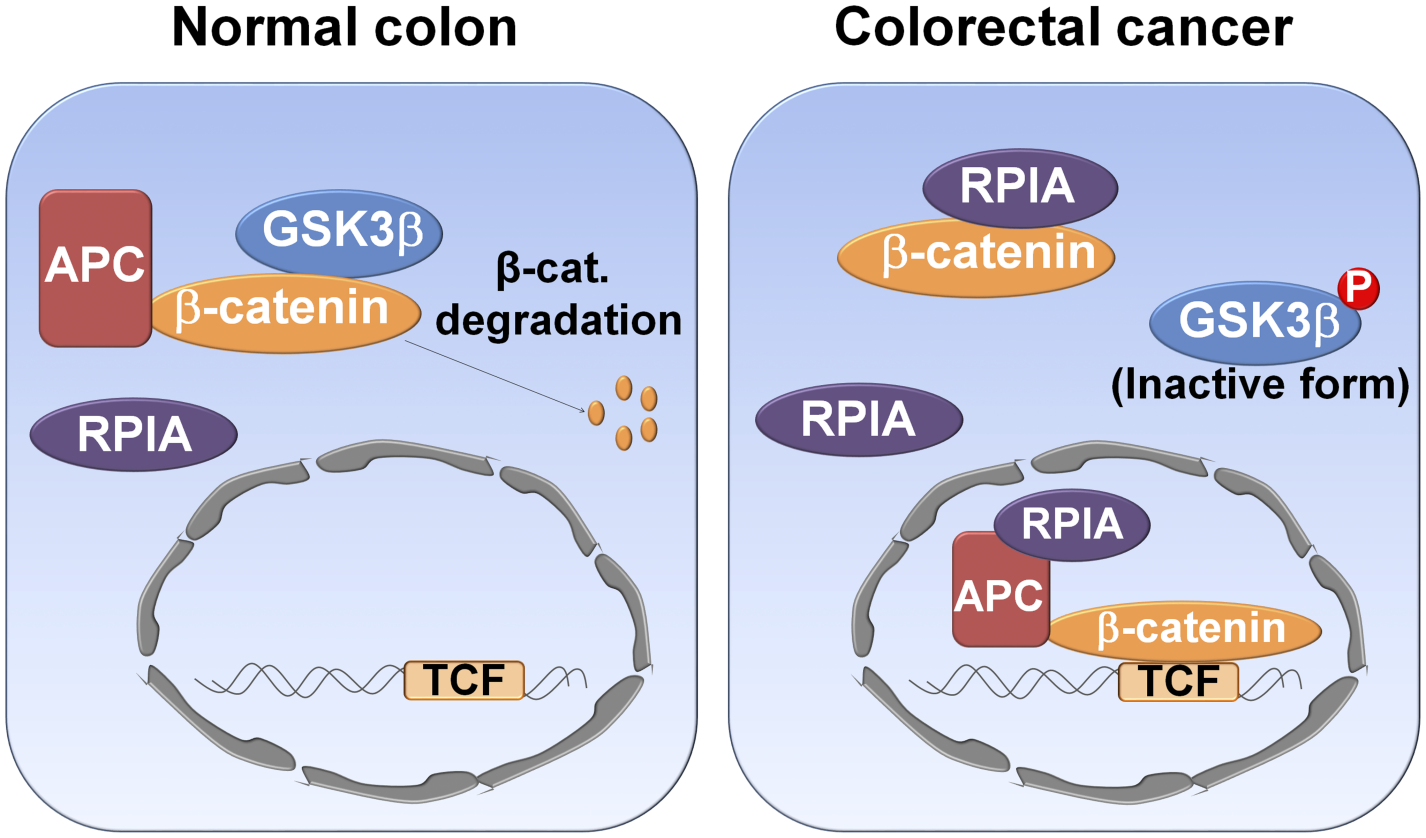
Model of the RPIA mechanism for induction of β-catenin signaling in CRCs. Schematic representation shows the role of RPIA and β-catenin signaling in normal and CRC cells. In normal cells, RPIA does not interfere with GSK3β-mediated β-catenin degradation. In CRC, RPIA is overexpressed in both the cytoplasm and the nucleus; RPIA then binds to β-catenin in cytoplasm, and this prevents the degradation of β-catenin mediated by GSK3β. Within the nucleus, increased levels of β-catenin protein might also be caused by RPIA interrupting the APC-mediated transport of β-catenin from the nucleus to the cytoplasm. Overall, this would result in activation of downstream β-catenin target genes. β-cat, β-catenin; APC, adenomatous polyposis coli; CRC, colorectal cancer; GSK3β, glycogen synthase kinase-3′; RPIA, ribose-5-phosphate isomerase A, TCF, T-cell transcription factor.

In the canonical β-catenin signaling pathway, APC binds to β-catenin in the cytoplasm in normal cells (Fig 7). This recruits GSK3β phosphorylates β-catenin, resulting in the eventual proteasomal degradation of β-catenin. We propose that in CRC cells, overexpression of RPIA results in the binding of RPIA to β-catenin and protects β-catenin from phosphorylation and subsequent cytoplasmic degradation. Intriguingly, we also found that RPIA interacts with APC and β-catenin in the nucleus. According to current studies, superfluous β-catenin is shuttled from the nucleus to the cytoplasm by APC [18,38]. We propose that RPIA might interrupt the APC-mediated process of exporting β-catenin by forming a complex in the nucleus. Consequently, colon cells developed tumorigenesis. Moreover, we found that C-terminal 22 amino acid of RPIA D domain is required for the RPIA-mediated β-catenin activation, stabilization, and enhanced colon cancer cell proliferation. This region is distinct from its enzymatic domain and a portion of the protein not previously identified as playing a role in CRC. Thus far, nothing is known about the function of the D domain, except this report. Cross species comparison of RPIA protein sequences between human, mouse, and zebrafish reveals that D domain is highly conserved across species. Accordingly, we hypothesize that the RPIA D domain exhibits a novel function in addition to the enzymatic region. It may associate with important partners, such as APC, β-catenin, and other proteins and form multimolecular complexes in both the cytosol and the nucleus. The phenomenon raises the questions such as “Does RPIA act as transcription co-activator?” and “Does RPIA have different protein partners in various cancer types?” We are currently searching for additional proteins that interact with RPIA in cancer cells.

The observation that RPIA is expressed at high levels early and throughout CRC is consistent with a role for RPIA in initiation and maintenance of carcinogenesis. Linking the clinical samples to our in vivo studies in zebrafish, misexpression of RPIA in the intestines of zebrafish is sufficient to induce spontaneous tumor formation in fish as young as 3 month and to cause additional physiological hallmarks of cancer, including reduced body weight, body width, and intestinal length in adult fish. These in vivo results are consistent with the observation that in cancer cell lines, downregulation of RPIA using microRNA reduces cell growth and colony formation ability, while overexpression of RPIA is correlated with enhanced growth and lower survival rates. In addition, the examination of apc/+ zebrafish revealed high levels of β-catenin that are disorganized and accumulate both in the cytoplasm and nucleus [45], and similar to our study, these fish develop spontaneous, intestinal tumors. In human patients, we noticed that RPIA expression was slightly decreased at the metastasis stage (Fig 1B and 1C). It was reported that invasive CRC cells exhibit low levels of proliferation markers [46]. We suggest that RPIA is necessary for primary tumorigenesis and that the RPIA level decreases at the metastasis stage so that tumor cells undergo epithelial-mesenchymal transition (EMT). Taken together, our studies demonstrate that RPIA functions as an activator for β-catenin-mediated colon tumorigenesis at the initiation stage.

One of the important functions of PPP is to generate ribose-5-phosphate for nucleotide synthesis. ATP provides the phosphate group via the salvage pathway [47], and RPIA mediates this enzymatic step in the cytoplasm. It has been proposed that knockdown of RPIA hinders tumor cell proliferation by reducing nucleotide synthesis [2]. However, in colorectal cells, the RPIA enzymatic and catalytic domain deletion mutants still promoted cell proliferation and activated β-catenin downstream target genes, revealing that at least in this type of cancer this is not how RPIA modulates tumor growth. In these cells, the RPIA D domain is necessary for cell proliferation, stabilization of β-catenin, and formation of a β-catenin/APC complex. Accordingly, D domain may be a therapeutic target for inhibiting the oncogenicity ability without affecting RPIA canonical enzymatic function in PPP. We therefore suggest that combination of anti-RPIA D domain therapy with conventional chemotherapy might improve the inhibition of CRC progression.

## Materials and methods

### Ethics statement

The mRNA from 80 paired tissues, including CRC and the adjacent normal tissues were obtained from Taipei Veterans General Hospital, procedures were undertaken in accordance with the Institutional Review Board of Taipei Veterans General Hospital, and the IRB number is 2015-04-010-AC. All adult participants provided written informed consent and there were no child participants.

All zebrafish experiments were approved by the Institutional Animal Care and Use Committee (IACUC) of the NHRI and were in accordance with International Association for the Study of Pain guidelines (protocol number: NHRI-IACUC-104157-A). Taiwan Zebrafish Core Facility (TZCF) at NHRI or TZeNH is a government-funded core facility, and since 2015, the TZeNH has been AAALAC accredited.

### Cell culture

The CRC cell lines HCT116 and SW480 were maintained in Dulbecco’s Modified Eagle’s Medium (DMEM) (Invitrogen, Carlsbad, CA) supplemented with 10% fetal bovine serum (FBS), 100 units/ml of penicillin, and 100 μg/ml of streptomycin and incubated at 37°C and 5% carbon dioxide. DNA typing of the cell lines was verified by Mission Biotech (Taipei, Taiwan) using a Promega GenePrint 10 System.

### Transfection and siRNA interference

Transient transfection of siRNA was performed using Lipofectamine RNAiMax (Invitrogen) according to the manufacturer’s manual. Three individual RPIA siRNAs (HSS117931, HSS117932, and HSS117933) and si-NC were purchased from Invitrogen.

### Plasmid construction and transfection

pcDNA3.0-RPIA, RPIA-ΔA, RPIA-ΔB, RPIA-Δ(A+B), RPIA-ΔC, and RPIA-ΔD were constructed by subcloning full-length or truncated RPIA cDNAs into a pcDNA 3.0 expression vector. Truncated RPIA products were amplified by specific primer sets: RPIA-ΔA (nt 1–522 deletion) (forward) 5ʹ ATAGAATTCATGGGCGGAGGCTG CCTGAC 3ʹ and (reverse) 5ʹ AGACTCGAGCTTGCAGGGTCAACAGAAAGGCT 3′; RPIA-ΔB (nt 523–567 deletion) (forward) 5ʹ ATAAGTCGCTTCATCGTGATCGCT 3ʹ and (reverse) 5ʹ AGAACCCTTGATGAGATTGAGATCA G 3′; RPIA-Δ(A+B) (nt 1–567 deletion) (forward) 5ʹ ATAGAATTCATGAGTCGCTTCATCGTGATCGCT 3ʹ and (reverse) 5ʹ AGACTCGAGCTTGCA GGGTCAACAGAAAGGCT 3′; RPIA-ΔC (nt 568–867 deletion) (forward) 5ʹ ATAATGGCTG AGAGAGTCTACTTTGGGATG 3ʹ and (reverse) 5ʹ AGAAGCATAGCCAGCCACAATCTTCT 3′; RPIA-ΔD (nt 868–936 deletion) (forward) 5ʹ ATAGAATTCACTTCAGCGGAGGCCGGAG 3ʹ and (reverse) 5ʹ AGACTCGAGGTTGATGAATAGGCCTGTGTCC 3ʹ. Transient transfection of the plasmid DNAs was performed using Lipofectamine 2000 (Invitrogen) according to the manufacturer’s manual.

### Hematoxylin-eosin (HE) and IHC staining

Colon cancer tissue staining data were obtained by using the tissue array CDA3 from SUPER BIO CHIPS and the tissue array MC5003a from US Biomax. The slides were incubated with mouse anti-RPIA (1:100) or rabbit anti-β-catenin (1:150) primary antibody at 4°C overnight after the dewax, rehydration, and antigen retrieval steps. The HE staining procedure was performed as outlined in our previous report, and tissues were examined with light microscopy [48].

### Western blotting, fractionation, and IP analysis

Total protein was extracted from cells using whole-cell extract (WCE) lysis buffer. Lysates were vibrated for 30 min and centrifuged at 13,200 rpm for 20 min at 4°C. Western blotting was performed as outlined in our previous report [24], and the fractionation protocol used has been described previously [49]. Primary antibodies include RPIA (Cat# ab67080; Abcam, Cambridge, United Kingdom), β-actin (Cat# GTX109639; GeneTex, Inc, Irvine, CA), β-catenin (Cat# GTX61089, GeneTex; Cat# ab22656, Abcam), β-catenin (phospho Ser33/Ser37) (Cat# GTX11350 GeneTex), APC (Cat# GTX61328, GeneTex), Ubiquitin (Cat# 3936; Cell Signaling Technology, Danvers, MA), K48-linkage specific polyubiquitin (Cat# 4289, Cell Signaling Tecnology), β-Tubulin (Cat# ab52866, Abcam), and Lamin A/C (Cat# ab108922, Abcam). For IP, 100 μg of protein lysate was incubated with primary antibody overnight and subsequently incubated with protein A/G-Sepharose beads for 1.5 h. The interaction results were assessed with western blotting.

### Reverse transcription and qPCR

RNA was extracted from paired samples of patient tissues, transgenic zebrafish tissue, or cell lines homogenized in TRIzol. cDNA was reverse transcribed from RNA using a High Capacity RNA-to-cDNA Kit (Cat# 4387406; Applied Biosystems, Foster City, CA). qPCR was performed using an ABI Prism 7500 Sequence Detection System (Power SYBR Master Mix, Cat#4367659, Applied Biosystems). Gene expression was amplified with the primers listed in Supporting information: S1 and S2 Tables.

### Luciferase reporter assay

Cells were transfected with TOPflash (containing a WT TCF binding site) or FOPflash (containing a mutated TCF binding site), which were purchased from Millipore, and *Renilla* luciferase was used as an internal control. The transfected cells were harvested 48 h post-transfection and lysed by the buffer supplied in the Dual-Glo Luciferase Assay Kit (Cat# E2940, Promega, Madison, WI), and luciferase activity in lysates was measured with a luminometer.

### Immunofluorescence assay

Transfected cells grown on cover slips were fixed in 4% paraformaldehyde for 10 min at room temperature and permeabilized in 0.5% Triton for 10 min. After 1 h of blocking in 2% FBS at room temperature, slides were incubated with anti-RPIA or anti-APC primary antibody at 4°C overnight. Secondary antibodies conjugated with Texas Red or FITC were used, and DAPI was used to stain nuclei. The images were scanned and captured with confocal microscopy.

### Transgenic zebrafish lines

The coding region of human *RPIA* (NM_144563.2) was amplified by PCR with the attB1-F-RPIA and attB2-R-RPIA primer pair using cDNA from the HEK293 cell line as a template. PCR was performed using a KOD FX (Toyobo, Osaka, Japan) and a 994-bp amplicon. The following forward primer was used for Gateway cloning: attB1-F-RPIA (Tm:59°C):^5ʹ^GGGGACAAGTTTGTACAAAAAAGCAGGCT**ATG**CAGCGCCCCGGGCC^3ʹ^, and the following reverse primer was used for Gateway cloning: attB2-R-RPIA (Tm:58°C):^5ʹ^GGGGACCACTTTGTACAAGAAAGCTGGGT**TCA**ACAGAAAGGCTTCTCCCTCATG^3ʹ^. PCR comprised the following steps: stage I: 94°C for 5 min; stage II (35 cycles): 95°C for 30 sec, 58°C for 30 sec, and 72°C for 2.5 min; stage III: 72°C for 7 min; and stage IV: 4°C.

Gateway cloning was performed to generate the final expression construct, namely, pTol2-ifabp: RPIA; myl7: EGFP, using a MultiSite Gateway Three-Fragment Vector Construction Kit (Invitrogen). The transgenic zebrafish model was established via microinjections of the above constructs, which were performed as described elsewhere, and the transgenic fish were selected as described previously [50]. The F2 generations of Tg (ifabp: RPIA; myl7: EGFP) zebrafish (*n* = 36) and AB line (WT) zebrafish which served as controls (*n* = 12) were analyzed in this study.

### Zebrafish husbandry

The zebrafish were maintained at the TZCF under an automated 14:10-h light:dark cycle and a constant temperature of 28°C under continuous flow. MS 222/tricaine methanesulfonate (160 mg/L) were applied for anesthesia.

## Supporting information

S1 Fig IRS standards of RPIA immunohistochemical staining. (A) The arbitrary IRS were calculated as compared to the IRS standards shown. Following RPIA IHC in multiple colon cancer tissue arrays (representing stage I, stage II, stage IIIB, stage IIIC, stage IVA, stage IVB, and metastatic) were graded for staining intensity as 1 (weak), 2 (moderate), and 3 (strong) and the percentage of positive cells were scored as 1 (<10%), 2 (11%–50%), 3 (51%–80%), and 4 (>80%). The IRS value is determined by the multiplication of the 2 different scores. Magnification: 200 X. Scale bar: 500 μm. (B) Three siRNA was designed from Invitrogen and pooled for using in colon cancer cell lines. The relative position of siRNA was shown. NCBI reference sequence of RPIA: NM_144563.2. IHC, immunohistochemistry; IRS, immunoreactive score; NCBI, National Center for Biotechnology Information; RPIA, ribose-5-phosphate isomerase A.
(TIF)

S2 Fig RPIA regulates colon cell proliferation through β-catenin expression in SW480 cells. (A) Knock down of RPIA significantly reduced cell proliferation, and RPIA overexpression enhanced cell proliferation in SW480 cells. Co-treatment of si-RPIA and pcDNA-RPIA rescued the reduction of cellular proliferation which upon knockdown of RPIA in SW480. Cell viability assays were performed by measuring the cells at the second, third, fourth, and fifth days as compared to the first day result of control cells. Control: Co-transfect with scramble RNA and pcDNA empty vector. (B) RPIA knockdown significantly reduced colony formation ability, and RPIA overexpression enhanced colony formation ability in SW480 cells. si-NC: Transfect with scramble siRNA as negative control. Representative images of the colonies were shown on top of the quantification result of colony formation. (C) Knockdown of RPIA reduced β-catenin protein levels as measured by western blotting (left panel) and quantified by image J (middle panel) but did not significantly alter mRNA levels of β-catenin as measured by qPCR (right panel) in SW480 cells. (D) RPIA overexpression increased β-catenin protein levels (left and middle panels) but did not affect β-catenin mRNA levels (right panel) in SW480 cells. (E) Knockdown of RPIA reduced the β-catenin/TCF luciferase reporter activity in SW480 cells. (F) Overexpression of RPIA induced the β-catenin/TCF luciferase reporter activity in SW480 cells. (G) Knockdown of RPIA decreased the mRNA levels of β-catenin target genes *CCND1* and *CCNE2* in SW480 cells. (H) Overexpression of RPIA increased the mRNA levels of β-catenin target genes *CCND1* and *CCNE2* in SW480 cells. The statistical significance was calculated by Student *t* test (** 0.001 < *P* < 0.01). Data can be found in S6 Data. *CCND1*, *Cyclin D1*; *CCNE2*, *Cyclin E2*; pcDNA, pcDNA vector control; qPCR, quantitative PCR; RPIA, ribose-5-phosphate isomerase A; si-NC, negative control siRNA; siRNA, small interfering RNA; si-RPIA, RPIA siRNA; TCF, T-cell transcription factor.
(TIF)

S3 Fig RPIA expression is positively correlated with β-catenin protein levels and stability in SW480 cells. (A) Knockdown of RPIA reduced β-catenin protein levels and RPIA overexpression increased β-catenin protein levels in both cytoplasmic and nuclear fractions of SW480 cells. (B) Knockdown of RPIA did not decrease ERK and pERK protein levels which were measured by western blotting in total protein analysis (up panel) in SW480. Conversely, overexpression of RPIA did not increase ERK and pERK protein levels (up panel). In the low panel, both cytoplasmic and nuclear fraction showed that ERK and pERK protein levels were not up-regulated in SW480. (C) Knockdown of RPIA did not decrease EGFR and pEGFR protein levels, which were measured by western blotting in total protein from HCT116 or SW480. (D) To determine the half-life of β-catenin protein, western blots were used to measure the abundance of β-catenin at different time points following the addition of the protein synthesis inhibitor CHX (10 μg/ml) in SW480 cells transfected with either control siRNA or RPIA-siRNA. The lower panels show plots of the relative β-catenin protein level, expressed as a percentage as a function of time after CHX treatment. (E) RPIA-ΔD lost the ability to stabilize β-catenin. Relative β-catenin protein levels as measured by quantification of western blot are shown in SW480 cells. (F) In SW480 cell, the reduced β-catenin levels by RPIA knockdown were rescued by 5 μM of MG132 treatment (left panel). Inhibition of RPIA stimulated ubiquitination of β-catenin (right panel). β-Catenin was precipitated by specific antibody. Coprecipitated ubiquitin levels were examined via western blot with anti-ubiquitin antibody. (G) The phosphorylated β-catenin (at Ser33/Ser37) versus total β-catenin was elevated upon RPIA knockdown. Gel images are shown on the up panel. (H) Overexpression of nondegradable β-catenin can overcome the growth inhibition by RPIA knockdown in SW480 cells. The proliferation fold is compared to pMCV6 transfected control cell at first day. (I) The elevated viability by expression of RPIA was decreased upon ICRT14 (β-catenin inhibitor) treatment. Dose-dependent effects were revealed in SW480. (J) pGSK3β^Ser9^ protein expression levels were up-regulated in cytoplasmic extraction upon overexpression of RPIA-WT, but not upon RPIA-ΔD in SW480 cells. (K) Cell proliferation was measured in RPIA knockdown in SW480 combined with 2.5 mM LiCl or 5 μM CHIR99021, respectively. The statistical significance was calculated by Student *t* test (* 0.01 < *P* < 0.05, ** 0.001 < *P* < 0.01, *** *P* < 0.001). Data can be found in S7 Data. CHX, cycloheximide; EGFR, epidermal growth factor receptor; ERK, extracellular signal-regulated kinase; LiCl, lithium chloride; pEGFR, phosphorylated-EGFR; pERK, phosphorylated-ERK; RPIA-ΔD, RPIA deletion domain D mutant; RPIA, ribose-5-phosphate isomerase A; RPIA-WT, RPIA wild type; siRNA, small interfering RNA.
(TIF)

S4 Fig RPIA localizes in nucleus and interacts with APC and β-catenin in SW480 cells. (A) Nuclear localization of RPIA was immunostained with an anti-RPIA antibody (green) in SW480 cells with and without overexpression of RPIA. DAPI was used to counterstain nuclei (blue). Scale bar: 50 μm. Co-localization of RPIA with APC or APC with β-catenin in SW480 were shown in fluorescence in the merge result. (B) Left panels: The cell lysates were precipitated by anti-APC, anti-β-catenin and anti-RPIA antibody in SW480 cells. The APC, β-catenin, and RPIA interaction can be increased by RPIA-WT but not by RPIA-ΔD. Right panels: Protein loading input for IP for SW480 cells. The orange boxes indicated those signals were enhanced by RPIA-WT but not in RPIA-ΔD. (C) Model of RPIA-β-catenin-APC interaction in SW480 cell line. APC, adenomatous polyposis coli; RPIA-ΔD, RPIA deletion domain D mutant; RPIA, ribose-5-phosphate isomerase A; RPIA-WT, RPIA wild type.
(TIF)

S5 Fig The mRNA and protein levels from WT and five deletion mutants, and the C-terminal domain of RPIA containing amino acid 290 to 311 is required for cell proliferation and β-catenin stabilization in SW480 cells. (A) The mRNA levels of WT and five deletion mutated-RPIA were analyzed by qPCR. (B) RPIA protein expression pattern was presented by western blot. The definite size is marked with asterisks. (C) The effect on cell proliferation after the expression of RPIA-WT and the different RPIA deleted constructs in SW480 cells. (D) RPIA-ΔD lost the ability to stimulate the TOPflash luciferase construct in SW480 cells. Data can be found in S8 Data. NS, no significant difference in statistics; qPCR, quantitative PCR; RPIA-ΔD, RPIA deletion domain D mutant; RPIA, ribose-5-phosphate isomerase A; RPIA-WT, RPIA wild type; WT, wild-type.
(TIF)

S6 Fig The expression level of β-catenin target genes was in 5-month-old and body weight, body width, intestine length and body length in 1-year-old RPIA Tg fish. The expression level of β-catenin target genes was analyzed in 5-month-old control fish (*n* = 6) and RPIA Tg fish (*n* = 18) from three portions of guts. The gene expression levels were analyzed with qPCR. There are extreme data in each group, so they are removed for the statistical analysis. (A) For IB, the number of WT is 5, and the number for RPIA is 17. (B) For MI, the number of WT is 3, and the number for RPIA is 18. (C) For PI, the number of WT is 5, and the number for RPIA is 18. One-year-old RPIA Tg fish (*n* = 21) and control fish (*n* = 18) were analyzed for the body weight, body width, intestine length, and body length (D-G). In the elder fish, overexpression of PRIA significantly decreased (D) body weight, (E) body width, and (F) intestine length (G). However, the body length was not affected. The statistical significance was calculated by Student *t* test (* 0.01 < *P* < 0.05, ** 0.001 < *P* < 0.01, *** *P* < 0.001). Data can be found in S9 Data. IB, intestinal bulb; MI, middle intestine; PI, posterior intestine; qPCR, quantitative PCR; RPIA, ribose-5-phosphate isomerase A; Tg, transgenic; WT, wild-type.
(TIF)

S1 Table The primer information for qPCR analysis in human cancer cell lines. This table lists the human qPCR primers. qPCR, quantitative PCR.
(DOCX)

S2 Table The primer information for qPCR analysis in RPIA transgenic zebrafish. This table lists the zebrafish qPCR primers. qPCR, quantitative PCR; RPIA, ribose-5-phosphate isomerase A.
(DOCX)

S1 Data Datasets to generate Fig 1A–1C. (XLSX)

S2 Data Datasets to generate Fig 2A–2H. (XLSX)

S3 Data Datasets to generate Fig 3A–3K. (XLSX)

S4 Data Datasets to generate Fig 5C and 5D. (XLSX)

S5 Data Datasets to generate Fig 6C, 6D and 6E. (XLSX)

S6 Data Datasets to generate S2 Fig. (XLSX)

S7 Data Datasets to generate S3 Fig. (XLSX)

S8 Data Datasets to generate S5 Fig. (XLSX)

S9 Data Datasets to generate S6 Fig. (XLSX)

## Abbreviations

APC: adenomatous polyposis coli
*CCND1*: *Cyclin D1*
*CCNE2*: *Cyclin E2*
CHX: cycloheximide
CRC: colorectal cancer
DMEM: Dulbecco’s Modified Eagle’s Medium
EGFR: epidermal growth factor receptor
EMT: epithelial-mesenchymal transition
ERK: extracellular signal-regulated kinase
FBS: fetal bovine serum
HCC: hepatocellular carcinoma
HE: hematoxylin-eosin
IACUC: Institutional Animal Care and Use Committee
IB: intestinal bulb
IHC: immunohistochemistry
IP: immunoprecipitation
IRS: immunoreactive score
LEF: lymphocyte enhancement factor
LiCl: lithium chloride
MI: middle intestine
pcDNA: pcDNA3 vector control
PDAC: pancreatic ductal adenocarcinoma
pEGFR: phosphorylated EGFR
pERK: phosphorylated ERK
PI: posterior intestine
PPP: pentose phosphate pathway
qPCR: quantitative PCR
RPIA: ribose-5-phosphate isomerase A
RPIA-WT: RPIA wild-type
RPIA-ΔD: RPIA deletion domain D mutant
si-NC: negative control siRNA
siRNA: small interfering RNA
si-RPIA: RPIA small interfering RNA
TCF: T-cell transcription factor
Tcf-4: T-cell transcription factor 4
Tg: transgenic
TZCF: Taiwan Zebrafish Core Facility
WCE: whole-cell extract
WT: wild-type
