# Supplementary figures and images for: Identification of a noncanonical function for ribose-5-phosphate isomerase A promotes colorectal cancer formation by stabilizing and activating β-catenin via a novel C-terminal domain
Source: PLoS Biol. 2018 Jan 16;16(1):e2003714. doi: 10.1371/journal.pbio.2003714 (PMC5786329; doi:10.1371/journal.pbio.2003714)

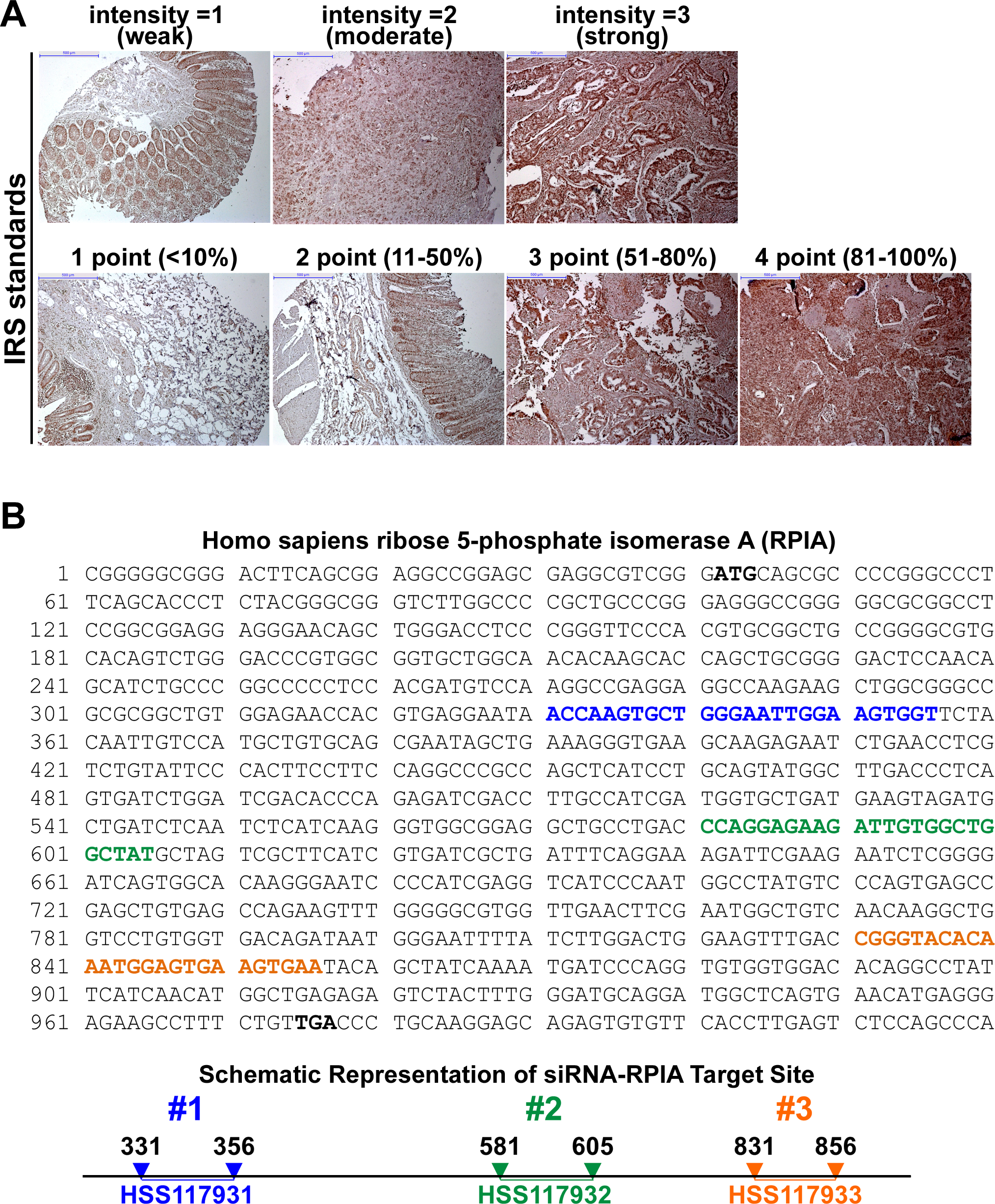

Supplement: S1 Fig — (A) The arbitrary IRS were calculated as compared to the IRS standards shown. Following RPIA IHC in multiple colon cancer tissue arrays (representing stage I, stage II, stage IIIB, stage IIIC, stage IVA, stage IVB, and metastatic) were graded for staining intensity as 1 (weak), 2 (moderate), and 3 (strong) and the percentage of positive cells were scored as 1 (<10%), 2 (11%–50%), 3 (51%–80%), and 4 (>80%). The IRS value is determined by the multiplication of the 2 different scores. Magnification: 200 X. Scale bar: 500 μm. (B) Three siRNA was designed from Invitrogen and pooled for using in colon cancer cell lines. The relative position of siRNA was shown. NCBI reference sequence of RPIA: NM_144563.2. IHC, immunohistochemistry; IRS, immunoreactive score; NCBI, National Center for Biotechnology Information; RPIA, ribose-5-phosphate isomerase A. (TIF) [file pbio.2003714.s001.tif]

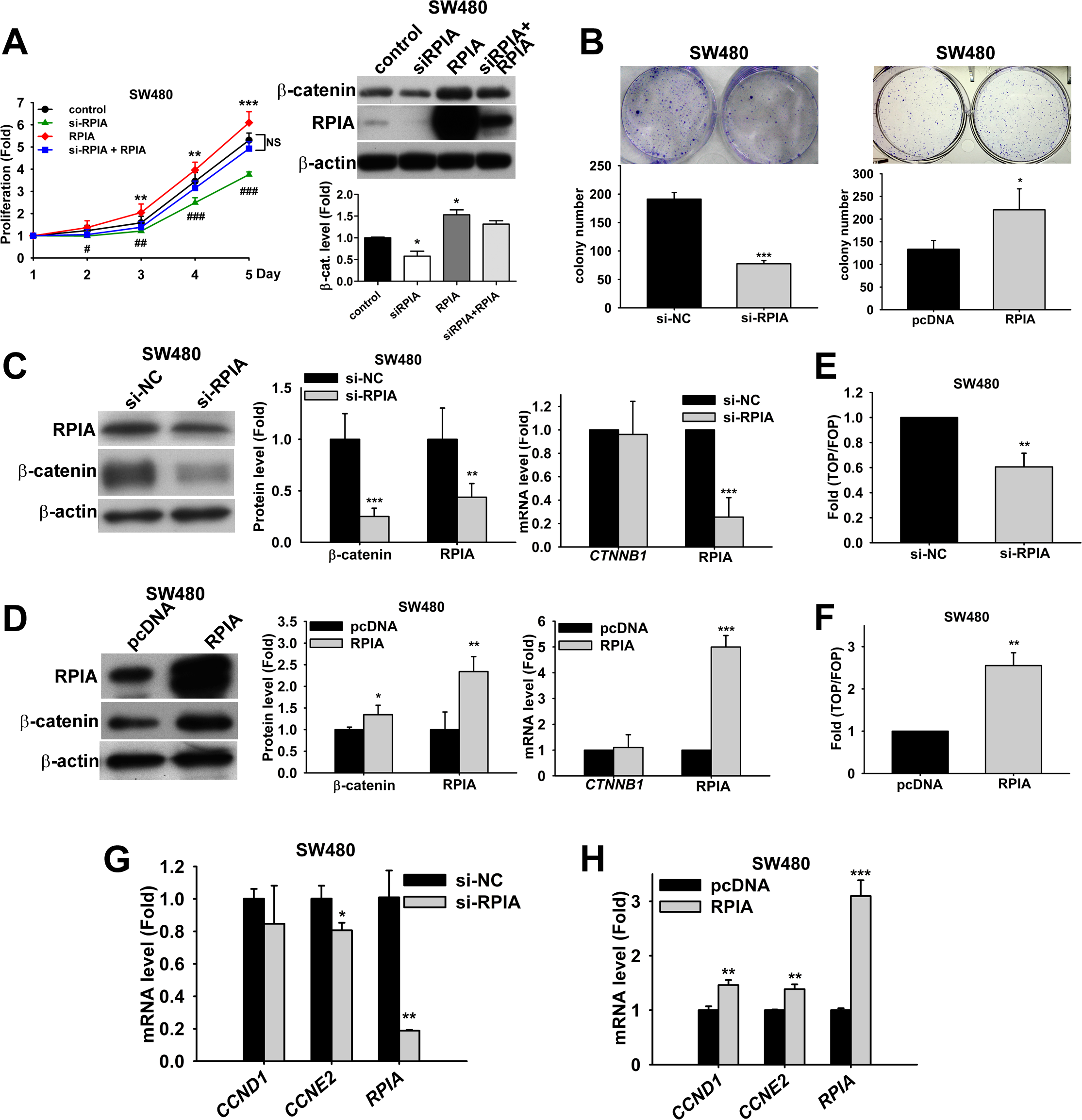

Supplement: S2 Fig — (A) Knock down of RPIA significantly reduced cell proliferation, and RPIA overexpression enhanced cell proliferation in SW480 cells. Co-treatment of si-RPIA and pcDNA-RPIA rescued the reduction of cellular proliferation which upon knockdown of RPIA in SW480. Cell viability assays were performed by measuring the cells at the second, third, fourth, and fifth days as compared to the first day result of control cells. Control: Co-transfect with scramble RNA and pcDNA empty vector. (B) RPIA knockdown significantly reduced colony formation ability, and RPIA overexpression enhanced colony formation ability in SW480 cells. si-NC: Transfect with scramble siRNA as negative control. Representative images of the colonies were shown on top of the quantification result of colony formation. (C) Knockdown of RPIA reduced β-catenin protein levels as measured by western blotting (left panel) and quantified by image J (middle panel) but did not significantly alter mRNA levels of β-catenin as measured by qPCR (right panel) in SW480 cells. (D) RPIA overexpression increased β-catenin protein levels (left and middle panels) but did not affect β-catenin mRNA levels (right panel) in SW480 cells. (E) Knockdown of RPIA reduced the β-catenin/TCF luciferase reporter activity in SW480 cells. (F) Overexpression of RPIA induced the β-catenin/TCF luciferase reporter activity in SW480 cells. (G) Knockdown of RPIA decreased the mRNA levels of β-catenin target genes CCND1 and CCNE2 in SW480 cells. (H) Overexpression of RPIA increased the mRNA levels of β-catenin target genes CCND1 and CCNE2 in SW480 cells. The statistical significance was calculated by Student t test (** 0.001 < P < 0.01). Data can be found in S6 Data. CCND1, Cyclin D1; CCNE2, Cyclin E2; pcDNA, pcDNA vector control; qPCR, quantitative PCR; RPIA, ribose-5-phosphate isomerase A; si-NC, negative control siRNA; siRNA, small interfering RNA; si-RPIA, RPIA siRNA; TCF, T-cell transcription factor. (TIF) [file pbio.2003714.s002.tif]

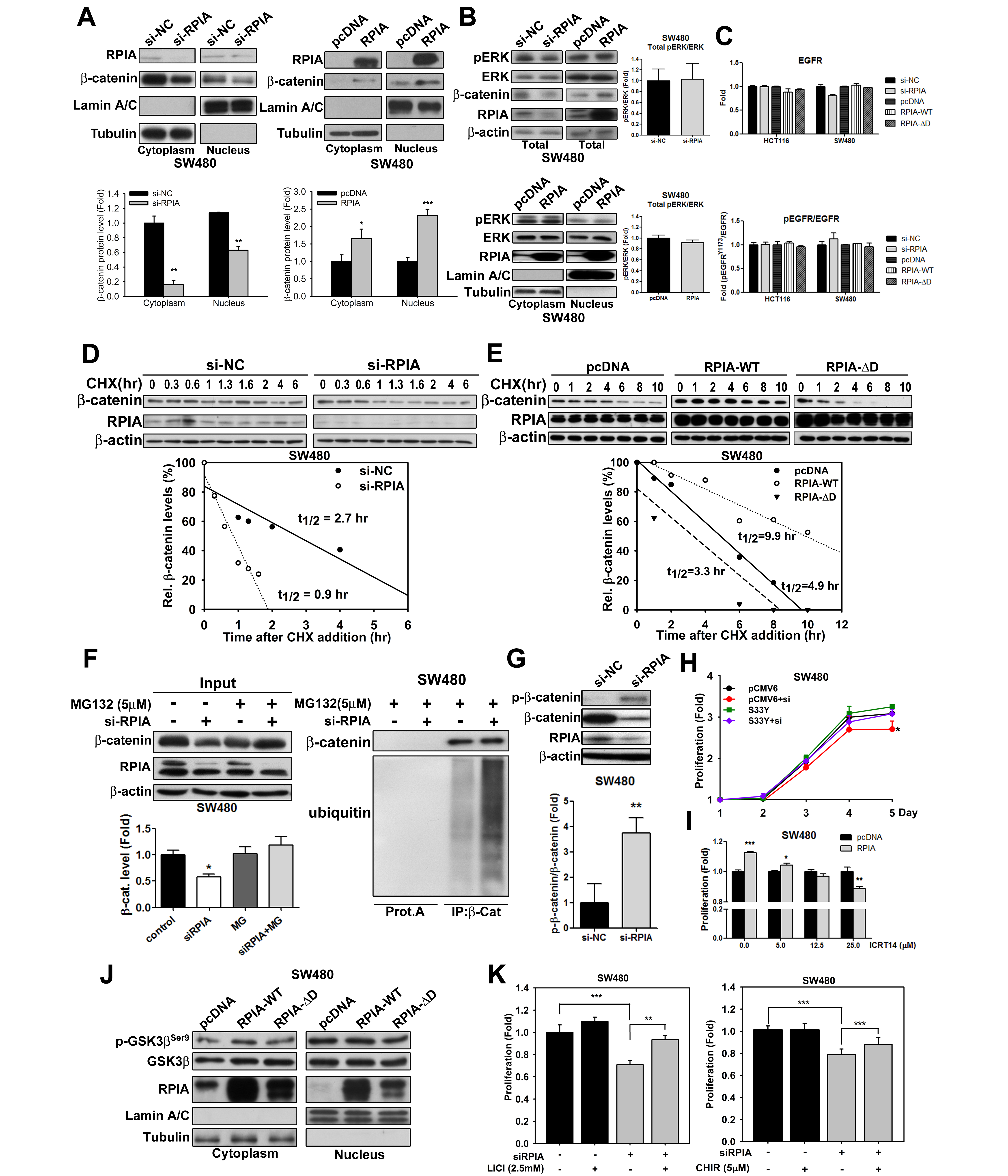

Supplement: S3 Fig — (A) Knockdown of RPIA reduced β-catenin protein levels and RPIA overexpression increased β-catenin protein levels in both cytoplasmic and nuclear fractions of SW480 cells. (B) Knockdown of RPIA did not decrease ERK and pERK protein levels which were measured by western blotting in total protein analysis (up panel) in SW480. Conversely, overexpression of RPIA did not increase ERK and pERK protein levels (up panel). In the low panel, both cytoplasmic and nuclear fraction showed that ERK and pERK protein levels were not up-regulated in SW480. (C) Knockdown of RPIA did not decrease EGFR and pEGFR protein levels, which were measured by western blotting in total protein from HCT116 or SW480. (D) To determine the half-life of β-catenin protein, western blots were used to measure the abundance of β-catenin at different time points following the addition of the protein synthesis inhibitor CHX (10 μg/ml) in SW480 cells transfected with either control siRNA or RPIA-siRNA. The lower panels show plots of the relative β-catenin protein level, expressed as a percentage as a function of time after CHX treatment. (E) RPIA-ΔD lost the ability to stabilize β-catenin. Relative β-catenin protein levels as measured by quantification of western blot are shown in SW480 cells. (F) In SW480 cell, the reduced β-catenin levels by RPIA knockdown were rescued by 5 μM of MG132 treatment (left panel). Inhibition of RPIA stimulated ubiquitination of β-catenin (right panel). β-Catenin was precipitated by specific antibody. Coprecipitated ubiquitin levels were examined via western blot with anti-ubiquitin antibody. (G) The phosphorylated β-catenin (at Ser33/Ser37) versus total β-catenin was elevated upon RPIA knockdown. Gel images are shown on the up panel. (H) Overexpression of nondegradable β-catenin can overcome the growth inhibition by RPIA knockdown in SW480 cells. The proliferation fold is compared to pMCV6 transfected control cell at first day. (I) The elevated viability by expression of RPIA [file pbio.2003714.s003.tif]

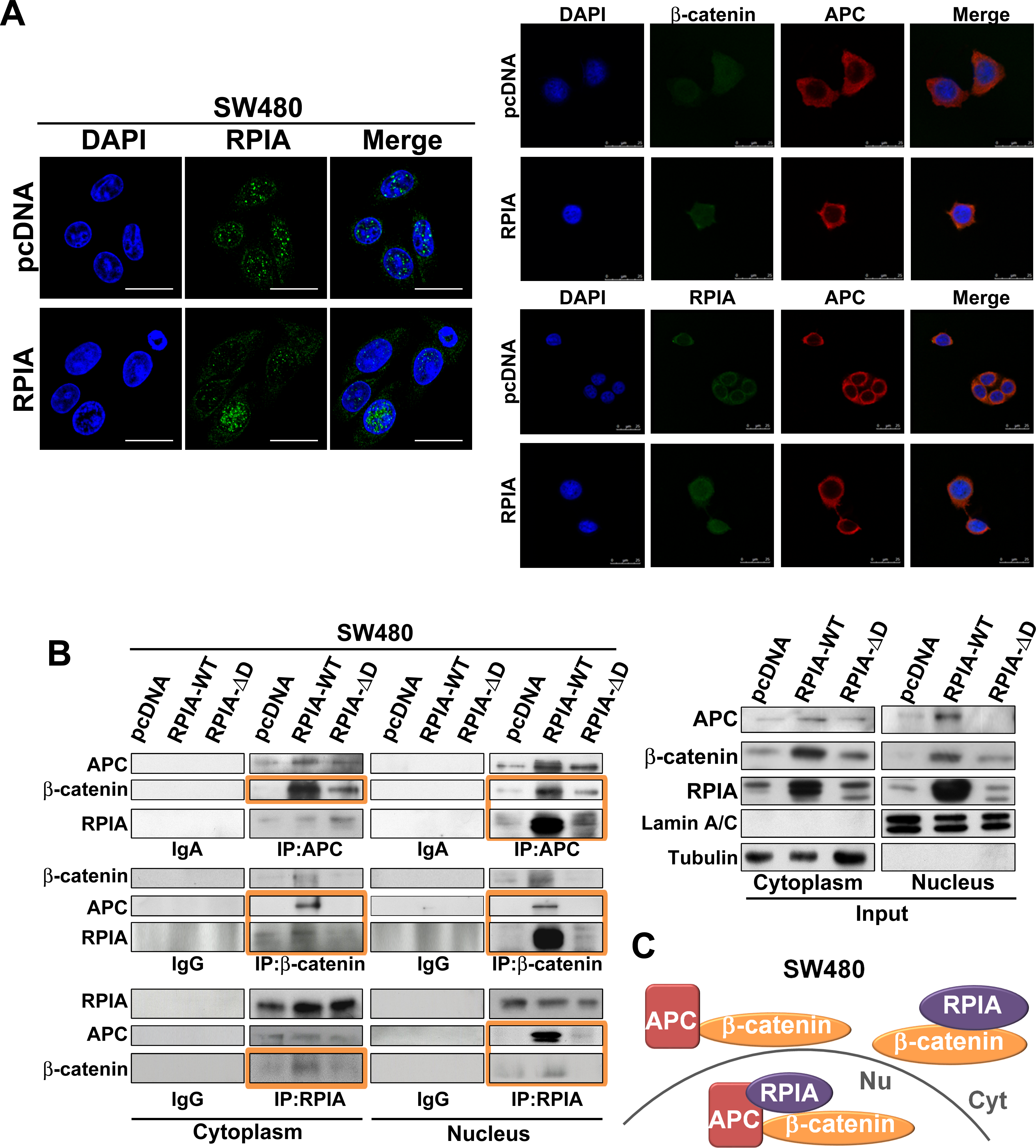

Supplement: S4 Fig — (A) Nuclear localization of RPIA was immunostained with an anti-RPIA antibody (green) in SW480 cells with and without overexpression of RPIA. DAPI was used to counterstain nuclei (blue). Scale bar: 50 μm. Co-localization of RPIA with APC or APC with β-catenin in SW480 were shown in fluorescence in the merge result. (B) Left panels: The cell lysates were precipitated by anti-APC, anti-β-catenin and anti-RPIA antibody in SW480 cells. The APC, β-catenin, and RPIA interaction can be increased by RPIA-WT but not by RPIA-ΔD. Right panels: Protein loading input for IP for SW480 cells. The orange boxes indicated those signals were enhanced by RPIA-WT but not in RPIA-ΔD. (C) Model of RPIA-β-catenin-APC interaction in SW480 cell line. APC, adenomatous polyposis coli; RPIA-ΔD, RPIA deletion domain D mutant; RPIA, ribose-5-phosphate isomerase A; RPIA-WT, RPIA wild type. (TIF) [file pbio.2003714.s004.tif]

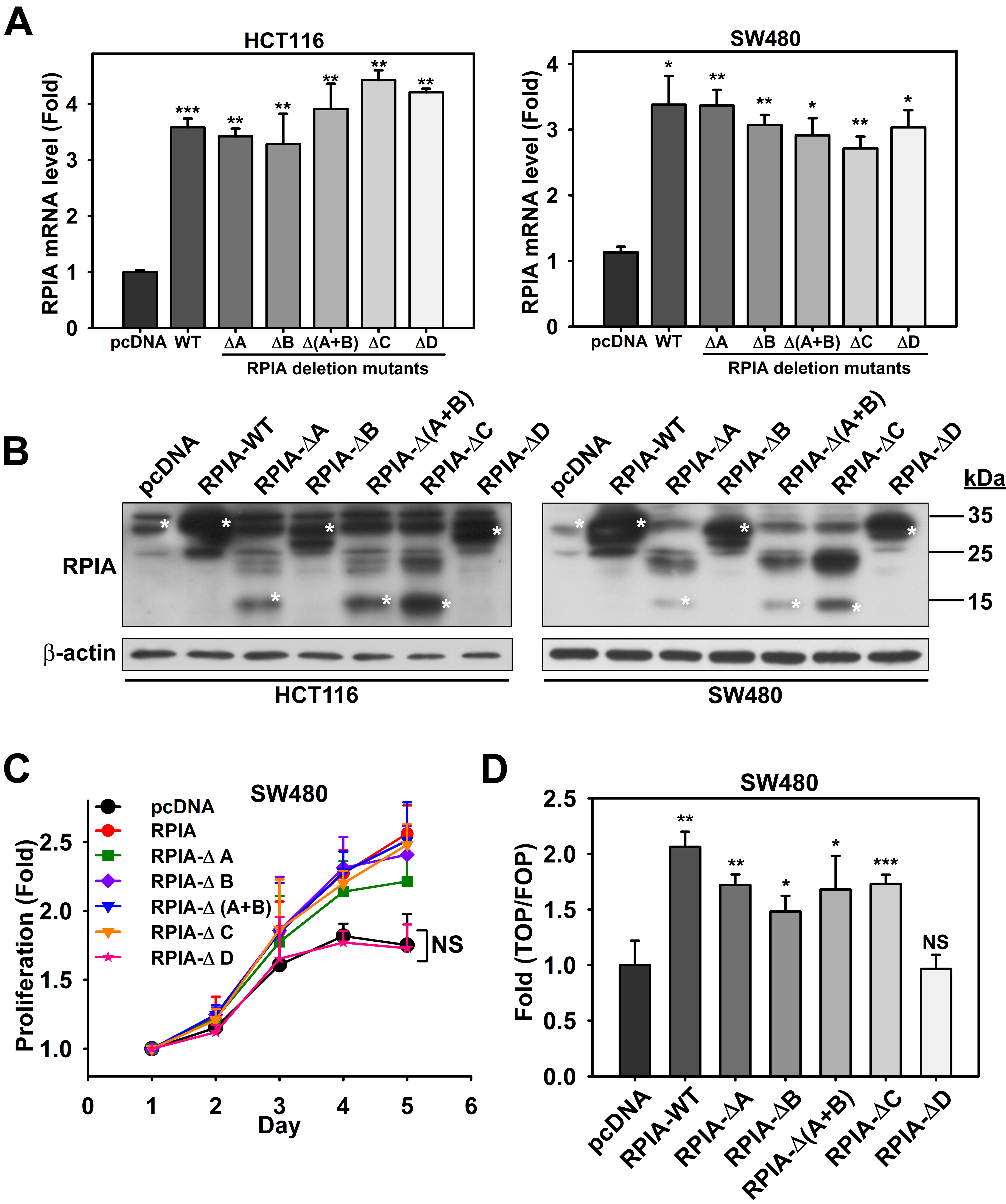

Supplement: S5 Fig — (A) The mRNA levels of WT and five deletion mutated-RPIA were analyzed by qPCR. (B) RPIA protein expression pattern was presented by western blot. The definite size is marked with asterisks. (C) The effect on cell proliferation after the expression of RPIA-WT and the different RPIA deleted constructs in SW480 cells. (D) RPIA-ΔD lost the ability to stimulate the TOPflash luciferase construct in SW480 cells. Data can be found in S8 Data. NS, no significant difference in statistics; qPCR, quantitative PCR; RPIA-ΔD, RPIA deletion domain D mutant; RPIA, ribose-5-phosphate isomerase A; RPIA-WT, RPIA wild type; WT, wild-type. (TIF) [file pbio.2003714.s005.TIF]

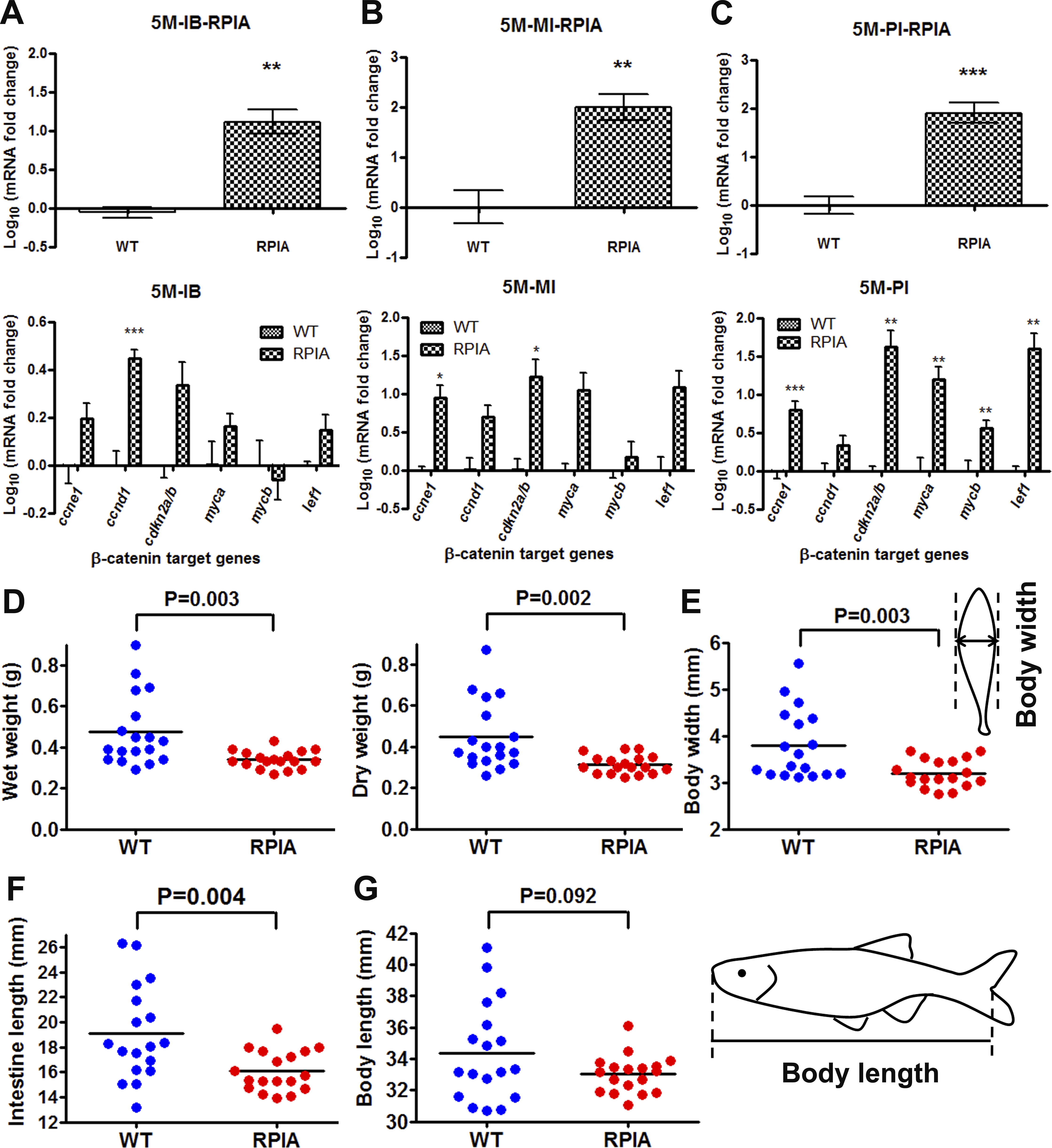

Supplement: S6 Fig — The expression level of β-catenin target genes was analyzed in 5-month-old control fish (n = 6) and RPIA Tg fish (n = 18) from three portions of guts. The gene expression levels were analyzed with qPCR. There are extreme data in each group, so they are removed for the statistical analysis. (A) For IB, the number of WT is 5, and the number for RPIA is 17. (B) For MI, the number of WT is 3, and the number for RPIA is 18. (C) For PI, the number of WT is 5, and the number for RPIA is 18. One-year-old RPIA Tg fish (n = 21) and control fish (n = 18) were analyzed for the body weight, body width, intestine length, and body length (D-G). In the elder fish, overexpression of PRIA significantly decreased (D) body weight, (E) body width, and (F) intestine length (G). However, the body length was not affected. The statistical significance was calculated by Student t test (* 0.01 < P < 0.05, ** 0.001 < P < 0.01, *** P < 0.001). Data can be found in S9 Data. IB, intestinal bulb; MI, middle intestine; PI, posterior intestine; qPCR, quantitative PCR; RPIA, ribose-5-phosphate isomerase A; Tg, transgenic; WT, wild-type. (TIF) [file pbio.2003714.s006.TIF]
