# Supplementary material for: Identification of a noncanonical function for ribose-5-phosphate isomerase A promotes colorectal cancer formation by stabilizing and activating β-catenin via a novel C-terminal domain
Source: PLoS Biol. 2018 Jan 16;16(1):e2003714. doi: 10.1371/journal.pbio.2003714 (PMC5786329; doi:10.1371/journal.pbio.2003714)
Supplement: S1 Table — This table lists the human qPCR primers. qPCR, quantitative PCR. (DOCX) [file pbio.2003714.s007.docx]

**S1 Table**

**The primer information for Q-PCR analysis in human cancer cell lines.**

| Target | Orientation | Primer sequence | location | Accession number |
| --- | --- | --- | --- | --- |
| *RPIA* | forward | 5' catgctgtgcagcgaatagc 3' | 369 -388 | NM_144563.2 |
|  | reverse | 5' TGGCGGGCCTGGAAGGAAGT 3' | 432 - 451 |  |
| *CTNNB1* | forward | 5' ACAAGCCACAAGATTACAAG 3' | 2262 - 2281 | NM_001904.3 |
|  | reverse | 5' ATCAGCAGTCTCATTCCAA 3' | 2335 - 2353 |  |
| *CCND1* | forward | 5' TCAAATGTGTGCAGAAGGAGGT 3' | 343 - 364 | NM_053056.2 |
|  | reverse | 5' GACAGGAAGCGGTCCAGGTA 3' | 459 - 478 |  |
| *AXIN2* | forward | 5' TGTGAGGTCCACGGAAACTG 3' | 1069 - 1088 | NM_004655.3 |
|  | reverse | 5' CGTCAGCGCATCACTGGATA 3' | 1194 - 1213 |  |
| *CCNE2* | forward | 5' CTATTTGGCTATGCTGGAGG 3' | 1190 - 1209 | NM_057749.2 |
|  | reverse | 5' TCTTCGGTGGTGTCATAATG 3' | 1271 - 1290 |  |
| *RNA18SN1* | forward | 5' ATGGCCGTTCTTAGTTGGTG 3' | 1332 - 1351 | NR_145820.1 |
|  | reverse | 5' CGCTGAGCCAGTCAGTGTAG 3' | 1529 - 1548 |  |
