# Supplementary material for: Identification of a noncanonical function for ribose-5-phosphate isomerase A promotes colorectal cancer formation by stabilizing and activating β-catenin via a novel C-terminal domain
Source: PLoS Biol. 2018 Jan 16;16(1):e2003714. doi: 10.1371/journal.pbio.2003714 (PMC5786329; doi:10.1371/journal.pbio.2003714)
Supplement: S2 Table — This table lists the zebrafish qPCR primers. qPCR, quantitative PCR; RPIA, ribose-5-phosphate isomerase A. (DOCX) [file pbio.2003714.s008.docx]

**S2 Table**

**The primer information for Q-PCR analysis in RPIA transgenic zebrafish.**

| Target | Orientation | Primer sequence | location | Accession number |
| --- | --- | --- | --- | --- |
| *ccne1* | forward | 5' CATGCCAAGCAAGAAAGTGCTA 3' | 191 - 212 | NM_130995.1 |
|  | reverse | 5' TGGGACGCACTGAAGTTGTTT 3' | 250 - 270 |  |
| *ccnd1* | forward | 5' TTGCCTCTCATCCCAGAACCT 3' | 945 - 965 | NM_131025.4 |
|  | reverse | 5' CCTGACACGATCGCAGACAGT 3' | 1423 - 1444 |  |
| *cdkn2a/b* | forward | 5' TTGACACCGTGCGGCTTT 3' | 1365 - 1382 | XM_002660468.5 |
|  | reverse | 5' CACATCCACTGGTCGTAAATCG 3' | 1423 - 1444 |  |
| *myca* | forward | 5' CACGCTGAAAGGAAGGAACTG 3' | 611 - 631 | NM_131412.1 |
|  | reverse | 5' GAGGTGCTCAGATCCTGCAAA 3' | 670 - 690 |  |
| mycb | forward | 5' CCTCAAGCGGTGTCACGTTA 3' | 934 - 953 | NM_200172.1 |
|  | reverse | 5' TGACTGCAGGCTGCTCATTC 3' | 994 - 1013 |  |
| *lef1* | forward | 5' CACGTTCCTACACCTGTCCCTAA 3' | 1855 - 1877 | NM_131426.1 |
|  | reverse | 5' AAGCAAGTGAGCGAGTGGACAT 3' | 1913 - 1934 |  |
| *actb1* | forward | 5' CTCCATCATGAAGTGCGACGT 3' | 893 - 913 | NM_131031.1 |
|  | reverse | 5' CAGACGGAGTATTTGCGCTCA 3' | 1052 - 1073 |  |
